# Novel strategies for mitigating hepatic ischemia-reperfusion injury: targeting MICU1 and calcium-glycolytic homeostasis with acteoside

**DOI:** 10.7150/ijbs.126332

**Published:** 2026-03-30

**Authors:** Yinhao Zhang, Runping Liu, Yun Yang, Huayao Lu, Mengyu Guo, Hong Wang, Ranyi Luo, Xiaoyong Xue, Guifang Fan, Kaihong Xie, Jia Liu, Xiaojiaoyang Li

**Affiliations:** 1 School of Life Sciences, Beijing University of Chinese Medicine, 100029, China.; 2 School of Chinese Materia Medica, Beijing University of Chinese Medicine, 100029, China.

**Keywords:** HIRI, LSEC, lactylation, calcium ion, acteoside, lipid nanoparticles

## Abstract

Hepatic ischemia-reperfusion injury (HIRI) contributes to metabolic disorders within hepatic sinusoid and frequently occurs during liver transplantation, yet its underlying mechanisms and intervention strategies remain obscure. This study aimed to elucidate whether acteoside (ACT) improved HIRI by repairing mitochondrial calcium uptake 1 (MICU1)-mediated Ca^2+^ dysregulation and facilitating glycolytic reprogramming. Using RNA sequencing, cleavage under targets & tagmentation (CUT&Tag) analysis, liver sinusoidal endothelial cells (LSECs)-specific overexpression virus or siMicu1 lipid nanoparticles and ACT derivatives, we explored the hepatoprotective mechanisms of ACT *in vivo* in HIRI mice and *in vitro* in hypoxia-reoxygenation or lactate-stimulated LSECs. ACT enhanced endoplasmic reticulum function and restored mitochondrial homeostasis, thereby alleviating LSECs damage and HIRI. Mechanistically, ACT directly bound to MICU1 and inhibited the overflow of Ca^2+^ from endoplasmic reticulum (ER) to mitochondria and subsequent mitochondrial Ca^2+^ overload. This competitive binding mode also suppressed MICU1-dependent glycolysis by blocking Ca^2+^-stimulated lactate production and histone H3K18 lactylation, which epigenetically regulated MICU1 transcription. Notably, ACT synergized with lactate inhibitors or *siMicu1* lipid nanoparticles to enhance its anti-HIRI effects, while LSECs-specific *Micu1* overexpression abolished these benefits. Structural analysis revealed that the C_26_/C_27_/C_40_/C_41_ hydroxyl groups determined ACT's MICU1-binding and hepatoprotective activities. This study identifies MICU1 as a central regulator of HIRI and reveals ACT as a targeted therapy by restoring Ca^2+^ balance and metabolic homeostasis.

## 1. Introduction

Hepatic ischemia-reperfusion injury (HIRI) is a significant contributor to early graft failure and liver dysfunction during or after liver transplantation and renders the new liver more susceptible to recurrent diseases, such as fibrosis and fatty liver diseases 1. Furthermore, the pathophysiology of HIRI is characterized by a biphasic response: ischemia-induced cell damage and metabolic disturbances and reperfusion-induced inflammatory responses. In the ischemic stage, hypoxia initially attracts hepatocytes, resulting in mitochondrial metabolic disorders, oxidative stress and subsequent different kinds of liver cell death within the hepatic sinusoids. Simultaneously, the injured or dead hepatic cells release reactive oxygen species (ROS) and damage-associated molecular patterns (DAMPs) that stimulate the loss of liver sinusoidal endothelial cells (LSECs) fenestrae and disrupt the immune regulatory interactions between hepatocytes and the circulatory system 2. The reperfusion phase further triggers the recruitment of neutrophils, macrophages and an inflammatory cascade reaction, leading to secondary liver damage and potentially multiple organ dysfunction 3, 4. To mitigate the significant clinical harm caused by HIRI and to address the current lack of established therapies or well-approved drugs, it is imperative to thoroughly dissect the mechanisms underlying HIRI and to develop new targeted intervention strategies.

Current research on the pathological mechanisms of HIRI primarily revolves around intracellular metabolic disorders, mitochondrial oxidative stress imbalance, and inflammatory damage. Our initial focus is to investigate whether additional complex regulatory patterns influence HIRI within the context of mitochondrial oxidative stress imbalance. Under hypoxic stimulation, the oxidative stress occurring within mitochondria may be modulated by other organelles. Recent studies have indicated that extensive endoplasmic reticulum (ER) stress induces the transfer of calcium ions (Ca^2+^) to mitochondria through specific protein channels, thereby promoting mtROS generation and initiating metabolic reprogramming characterized by enhanced glycolytic flux and excessive lactate accumulation 5. Here, we notice a significant damage-inducing factor, lactate, which is a normal intracellular metabolite that also serves as a substrate and regulator for epigenetic modifying enzymes. Recently, elevated levels of lactate were reported to influence histone activity through a newly identified epigenetic modification mechanism termed histone lactylation. This mechanism entailed the addition of lactyl groups to lysine residues on histones, thereby facilitating epigenetic modifications and modulating the transcriptional responses of genes associated with energy metabolism and tissue repair 6. Meanwhile, recent studies have consistently confirmed the regulatory role of histone lactylation in liver fibrosis 7, glioma 8 and neurodegenerative diseases 9. However, it remains undetermined whether histone lactylation modification contributes to HIRI-related transcriptional activation and interacts with Ca^2+^ imbalance-driven metabolic reprogramming and epigenetic regulation.

Acteoside (ACT, CAS: 61276-17-3) is a natural phenylpropanoid glycoside derived from *Rehmanniae Radix Praeparata* root, *Cistanches Herba* stems and *Herba Plantaginis* seeds, recognized for its ability to alleviate hepatocyte apoptosis, oxidative stress and exert anti-inflammation or anti-cancer properties. Among the diverse intrahepatic cell types, LSECs represent a unique yet often overlooked population that sense external damage stimuli and influence other hepatic cells by releasing liver metabolism-related molecules through their “fenestrate” structures 2. Recently, we first demonstrated that ACT reversed the senescent phenotype of LSECs, reduced the transfer of high-mobility group box 1 protein (HMGB1) from hepatocytes to LSECs and thereby alleviated HIRI 10. Meanwhile, we further noticed that ACT effectively mitigated the hepatic ferroptosis process in HIRI by suppressing the poly (rC)-binding protein 2 (PCBP2)-hypoxia-inducible factor 1α (HIF1α)-HMGB1 axis 11. Although there is no evidence suggesting a direct correlation between ACT and the regulation of histone lactylation, recent studies have confirmed that lactate can regulate the lactylation of direct ACT targets like HMGB1 in macrophages 12. However, the causal relationship among histone lactylation modification, Ca^2+^ imbalance-driven mitochondrial homeostasis and ACT-mediated protection against HIRI still requires further validation.

Here, we first demonstrated that ACT directly targeted the Ca^2+^ signaling hub mitochondrial calcium uptake 1 (*Micu1*, MICU1) to prevent Ca^2+^ overload, thereby maintaining the homeostasis of the ER-mitochondrial axis and protecting HIRI through a dual pathway. Firstly, by competitively binding to the MICU1 protein, ACT temporally blocked the abnormal transfer of Ca^2+^ from the ER to the mitochondria. Secondly, by inhibiting histone H3 lysine 18 lactylation (H3K18la) modification, ACT epigenetically downregulated the transcription of the *Micu1*, thereby reducing the risk of Ca^2+^ overload at its source. This study reveals a novel mechanism by which ACT, through the dual pathway of metabolic microenvironment remodeling and epigenetic modification intervention, disrupts the vicious cycle of Ca^2+^ imbalance and ameliorates HIRI, providing a new perspective for HIRI intervention.

## 2. Materials and Methods

### 2.1 Preparation and characterization of lipid nanoparticles (LNPs)-siMicu1

LNPs entrapping siMicu1 were designed for efficient liver targeting. Briefly, fluorescein isothiocyanate (FITC) was first covalently conjugated to hyaluronic acid (HA) *via* EDC/NHS-mediated coupling to generate FITC-labeled HA derivatives. Cholesterol and DSPE-PEG were then dissolved in ethanol, and 0.1 mol% FITC-labeled HA derivatives were added to the lipid mixture. Subsequently, a T-junction mixer was used to quickly mix siMicu1 and above solution, which was then dialyzed, filtered and concentrated overnight to obtain the final preparation of LNPs-siMicu1 (100 μl suspension in 10 ml PBS).

For physicochemical characterization of LNPs-siMicu1, we conducted dynamic light scattering measurements to assess the polydispersity index (PDI) and zeta potential values of LNPs-siMicu1, utilizing an immersion cell electrode (ZEN1002, Malvern) in conjunction with a Zetasizer (Nano ZS, Malvern Co.). All reported zeta potentials were derived from triplicate measurements. Additionally, we investigated the morphology of LNPs-siMicu1 through negative stain imaging using transmission electron microscopy (JEM 1200EX, JEOL). The encapsulation efficiency of siMicu1 was determined using high-performance liquid chromatography (HPLC). Specifically, we employed ultrasonic disruption or high-speed centrifugation of LNPs-siMicu1 to isolate total LNPs or free siMicu1. The HPLC analysis was performed on an UltiMate 3000 liquid chromatograph (Thermo Scientific, Sunnyvale), utilizing a Welch Ultimate^TM^ C18 column (250 mm × 4.6 mm; 5 μm). The mobile phase consisted of ultrapure water, acetonitrile, and methanol in a ratio of 39%: 37%: 24%. The encapsulation efficiency (EE%) = (Amount of encapsulated siMicu1 / Total initial amount of siMicu1) × 100%. To evaluate the *in vivo* targeting efficacy of LNPs, we prepared FITC-labeled LNPs-siMicu1 and administered them to mice *via* tail vein injection. The IVIS *in vivo* imaging system (PerkinElmer, Hopkinton) was employed to capture fluorescence signal images of living mice and various organs (heart, liver, spleen, lung and kidney) at 0, 2, 4 and 6 h post-injection.

### 2.2 Construction of Micu1-overexpressing (oeMicu1) AAV8 virus targeting LSECs

The stabilin 2 (*Stab2*) promoter has been previously demonstrated to exhibit high LSECs specificity* in vivo* and is widely used as a reliable endothelial-targeting regulatory element [PMID:38333675]. AAV vectors were constructed for efficient LSECs targeting. The initial plasmid contained a cytomegalovirus (CMV) promoter, green fluorescent protein (GFP) and Flag gene. The restriction sites for *Bsu15I* and *EcoRI* were introduced at the termini of the *Stab2* (500-144 bp) promoter fragment. Th*e Stab2* (500-144 bp)-Flag-GFP plasmid was created by replacing the CMV promoter region in the CMV-Flag-GFP plasmid with the *Stab2* (500-144 bp) promoter fragment. Subsequently, the *Micu1* gene fragment was cloned into the *Stab2* (500-144 bp)-Flag-GFP plasmid using the HB infusion^TM^ ligation system, resulting in the *Stab2* (500-144 bp)-Micu1-Flag-GFP plasmid. The CMV-GFP plasmid served as a control. All constructed plasmids were verified through restriction enzyme digestion analysis and DNA sequencing.

For AAV8 packaging and preparation, after high-purity endotoxin-free extraction, we used Lipofiter^TM^ transfection reagent (Hanheng, China) to co-transfect AAV-293 cells with Stab2 (500-144 bp)-Micu1-Flag-GFP plasmid, RC plasmid and pHelper plasmid. Cell pellets were collected 72 h after transfection. After collecting the supernatant containing AAV and performing column purification, we obtained a high-titer adeno-associated virus preservation solution. The SYBR Green method was then employed to quantify the AAV genome content. The mean Ct value of each group of AAV standards was plotted on the ordinate (Y-axis), while the logarithm of the corresponding copy number was plotted on the abscissa (X-axis), yielding a standard curve (*y* = -2.851*x* + 32.296, R^2^ = 0.9957). The AAV genome titer (vector genomes per milliliter, vg/ml) was determined by quantitative PCR against the GFP sequence. The copy number was calculated from the standard curve and the final titer was derived by multiplying by the corresponding dilution factors. The final titers for the target virus and the control virus were determined to be 1.6 × 10^12^ vg/ml and 1.3 × 10^12^ vg/ml, respectively. All prepared AAV8 were subpackaged and stored at -80 °C until needed.

### 2.3 Synthesis and identification of ACT derivatives

To replace all hydroxyl groups in ACT, we employed a heating reflux method involving ACT and acetic anhydride. Specifically, 200 mg of ACT was combined with 10 ml of acetic anhydride, stirred and heated to reflux for 3 h. Subsequently, 20 ml of DCM was added to the refluxed mixture, which was then extracted with water several times. The aqueous layer was discarded, and the DCM layer was concentrated under reduced pressure. To eliminate the active structure of catechol moieties, we followed the ACT hydrolysis protocol under alkaline conditions. Specifically, we utilized two equivalents (2.0 equiv.) of LiOH in relation to ACT as a base, hydrolyzed ACT for 4 h at 37 °C, and subsequently extracted the product using DCM. To identify the obtained ACT derivatives, we performed UPLC-UV analysis using an UltiMate 3000 liquid chromatography system (Thermo Scientific, Sunnyvale, CA, USA). The separation was carried out on a Welch Ultimate^TM^ C18 column (250 mm × 4.6 mm; 5 μm) with mobile phase A consisting of 0.1% formic acid in water and mobile phase B being acetonitrile. The gradient elution program was set as follows: 0-1 min, 5% B; 1-25 min linear gradient from 5% to 60% B; 25-30 min gradient from 60% to 90% B. The total run time was 30 minutes with a flow rate of 0.2 ml/min, injection volume of 10 μl, and full wavelength detection. Mass spectrometry analysis was conducted using a Q Exactive hybrid quadrupole-Orbitrap mass spectrometer (Thermo Scientific, Bremen, Germany) for data acquisition. The ionization parameters were configured with a capillary temperature of 320 °C, spray voltage of 3.5 kV, and auxiliary gas heater temperature of 400 °C. Full-scan analyses were performed in both positive and negative ionization modes, covering a mass range of m/z 100-1500.

### 2.4 Animal studies

C57BL/6J mice (8 weeks old, male, 22-24 g) were procured from SIBEIFU Biotechnology Co. (Beijing, China). All mice were housed in a temperature and diurnal light (12 h light/12 h dark cycle) controlled environment (22 ± 2 °C) with access to standard chow and sterile water. All animal care and experimental procedures were approved by the Institutional Animal Protection and Use Committee of Beijing University of Traditional Chinese Medicine (BUCM-2024112902-4191). Animal studies were reported in compliance with the ARRIVE guidelines.

**Figure 1:**
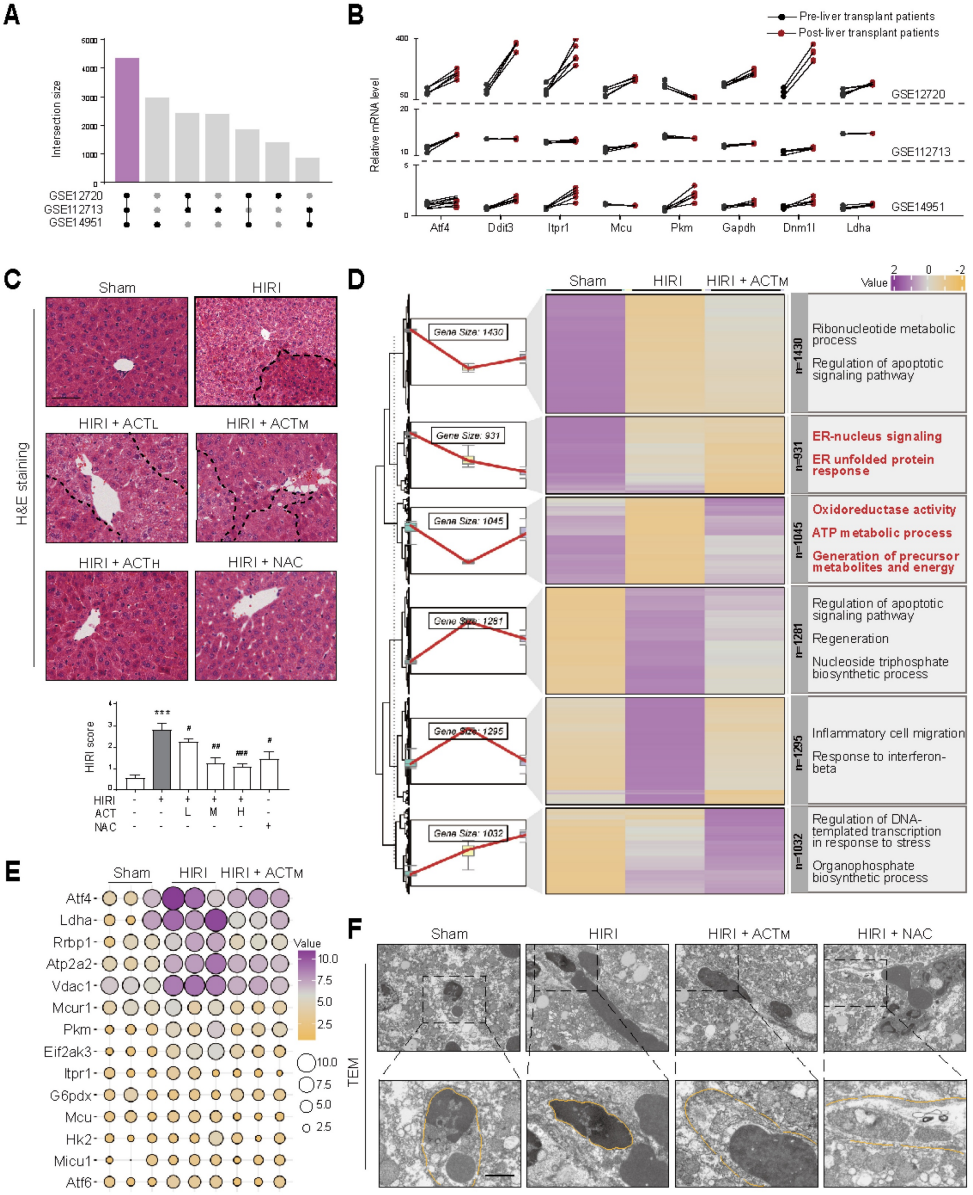
** ACT ameliorates HIRI through coordinated regulation of ER stress and mitochondrial bioenergetics** (**A**) Venn analysis of DEGs in the human pre-HIRI and post-HIRI liver tissues. (**B**) Gene expression of ER stress and mitochondrial respiration regulators in paired human pre-HIRI *vs.* post-HIRI groups. ACT was administered intraperitoneally at 25, 50 and 100 mg/kg (i.p.) for 7 consecutive days prior to HIRI surgery. (**C**) Representative H&E staining photomicrographs, scale bar = 100 μm. (**D**) Hierarchical clustering of transcriptomic profiles in Sham, HIRI and HIRI+ACT groups. (**E**) Bubble maps highlighting core genes in ER stress response and mitochondrial respiratory chain processes based on liver RNA-seq data. Gene expression levels were calculated using FPKM values. The color gradient indicated relative gene expression levels, while the bubble size reflected the expression abundance across different experimental groups. (**F**) Representative images of TEM in the liver, scale bar = 5 μm. Data were presented as mean ± SEM (n = 6 for mice). ***P < 0.001, compared to the sham group; ^#^*P* < 0.05, ^##^*P* < 0.01, ^###^*P* < 0.001, compared to the HIRI group by two-way ANOVA (**C**). See also **Figure S1**. ACT, acteoside; *Atf4*, activating transcription factor 4; *Atp2a2*, ATPase sarcoplasmic/endoplasmic reticulum Ca^2+^ transporting 2; *Ddit3*, DNA damage inducible transcript 3; *Dnm1l*, dynamin 1 like; *Eif2ak3*, eukaryotic translation initiation factor 2 alpha kinase 3; ER, endoplasmic reticulum; G6pdx, glucose-6-phosphate dehydrogenase; *Gapdh*, glyceraldehyde-3-phosphate dehydrogenase; H&E, hematoxylin and eosin; HIRI, hepatic ischemia-reperfusion injury; *Hk2*, hexokinase 2; *Itpr1*, inositol 1,4,5-trisphosphate receptor type 1; *Ldha*, lactate dehydrogenase A; *Mcu*, mitochondrial calcium uniporter; *Mcur1*, mitochondrial calcium uniporter regulator 1; *Micu1*, mitochondrial calcium uptake 1; NAC, N-acetylcysteine; *Pkm*, pyruvate kinase M; *Rrbp1*, ribosome binding protein 1; TEM, transmission electron microscopy; *Vdac1*, voltage-dependent anion channel 1.

For the animal experiments in **Figure 1**, mice were randomly assigned to six groups (n = 6 mice per group): (1) Sham group; (2) HIRI group; (3) HIRI + ACT low-dose (L, 25 mg/kg) group; (4) HIRI + ACT medium-dose (M, 50 mg/kg) group; (5) HIRI + ACT high-dose (H, 100 mg/kg) group; (6) HIRI + N-Acetyl-L-cysteine (NAC, 100 mg/kg) group. Given that ACT is a water-soluble glycoside compound, oral administration is susceptible to degradation through first-pass metabolism. Therefore, ACT was administered intraperitoneally at 25, 50 and 100 mg/kg, with NAC (100 mg/kg) as a positive control, based on our previous research 10. Mice in groups 1 and 2 received an intraperitoneal injection of 10 ml/kg saline as a parallel control, while mice in groups 3-6 were administered the corresponding drug *via* intraperitoneal injection within one week prior to surgery. Subsequently, HIRI surgery was performed on mice in groups 2 to 6 11. Briefly, after ACT pretreatment, mice were anesthetized with isoflurane, and the liver was exposed through a midline laparotomy. Noninvasive microvascular forceps were utilized to clamp the portal vein and hepatic artery for 60 min and released for 6 h of reperfusion 13. The mice in the sham group underwent the same surgical procedure without vessel clamping and release. At the end of procedures, all livers and serum samples were collected immediately for further analysis 14.

For the animal experiments in **Figure 7**, mice were randomly assigned to four groups (n = 6 mice per group): (1) Sham group; (2) HIRI group; (3) HIRI + LNPs-siMicu1 group; (4) HIRI + LNPs-siMicu1 + ACT (50 mg/kg) group. Mice in groups 3 and 4 were received an intravenous injection of LNPs-siMicu1 (5 μl/g) 2 h prior to surgery. ACT administration and HIRI procedures were performed using the same protocol as described above.

For the animal experiments in **Figure 8**, mice were randomly assigned to four groups (n = 6 mice per group): (1) Sham group; (2) HIRI group; (3) HIRI + AAV8-stab2-oeMicu1 group; (4) HIRI + AAV8-stab2-oeMicu1 + ACT (50 mg/kg) group. Mice in groups 3-4 were intravenously injected with AAV-oeMicu1 (5 μl/g) 14 days before surgery. Other surgical manipulations and drug administration protocols in these studies were the same as the first animal batch.

### 2.5 Cell culture and treatment

We previously constructed a two-step perfusion method to isolate primary mouse LSECs follow by two Percoll gradient separations 15. Primary LSECs, LSECs (BNBIO) line and HEK293T cell lines were cultured at 37 °C in a 5% CO2 atmosphere using DMEM supplemented with 10% fetal bovine serum and 1% penicillin G-streptomycin (100 U/ml penicillin and 100 µg/ml streptomycin). Cells were pretreated with DMSO or ACT (25, 50 and 100 μM) for 8 h, subjected to hypoxic for 16 h, and then reoxygenated for 8 h (hypoxia-reoxygenation, HR). ACT concentrations were selected based on reported dosages and our previous studies 10, 16, 17. In the lactate accumulation model, LSECs were treated with lactate (5 mM) for 24 h according to established protocols. For cell transfection, LSECs were transfected with Micu1-siRNA using Lipofectamine RNAi MAX transfection reagent (Hanheng, China) for 48 h, incubated with ACT in Opti-MEM medium for 1 h and then established HR or lactate models for another 20 h. In the HR or lactate-accumulation model, the inhibition of lactate or Ca^2+^ were achieved by administering 5 mM oxamate (OM) or 10 μM galloflavin (GF) for 24 h, or 100 μM 2-aminoethyl diphenylborinate (2-APB) for 8 h following ACT pre-administration.

### 2.6 RNA sequencing (RNA-Seq) of liver and LSECs

For bulk liver-seq, we extracted total RNA from mouse liver using Trizol reagent and quantifying RNA concentration using Nano Rhatometer@Spectrophotometer (IMPLEN, USA). Briefly, after purifying mRNA and enriching 250-300 bp cDNA fragments, we constructed a sequencing library on the Illumina Novaseq platform. For single-cell type RNA-seq, we used the Trizol classic extraction method to extract total RNA from primary LSECs. After evaluating the RNA purity, concentration and integrity by a NanoRhatometer spectrophotometer (IMPLEN, USA), we prepared primary LSECs RNA libraries and sequenced the indexed libraries on an Illumina NovaSeq 6,000 (2 x 150 bp). Based on STRING (https://string-db.org), Kyoto Encyclopedia of Genes and Genomes (KEGG) (https://www.kegg.jp) and DAVID database (https://david.ncifcrf.gov), we used ggplot2 in R to perform enrichment analysis of gene ontology (GO) and KEGG pathway in R to analyze correlations between genes.

### 2.7 Cleavage under targets and tagmentation (CUT&Tag) experiment

After different treatments, cells were centrifugation at 1,200 rpm for 3 min, a total of 100,000 LSECs per sample were subjected to CUT&Tag. Briefly, we incubated LSECs with NE1 buffer (20 mM HEPES, pH 7.9, 10 mM KCl, 0.1% Triton X-100, 20% glycerol, 0.5 mM spermidine and EDTA-free protease inhibitors) on ice for 10 min. After collecting the nuclear pellet, concanavalin A-coated magnetic beads (ConA beads) were added to the resuspended cells and incubated to facilitate cell binding. We subsequently used the nonionic detergent 0.01% digitonin to remove the unbound supernatant and incubated cells with ConA beads containing H3K18la antibody, a secondary antibody, and Hyperactive pA-Tn5 transposase. The active pA-Tn5 transposase cleaved DNA fragments associated with the target protein, which were then ligated to P5 and P7 adapters. Additionally, we added 2 μl of universal i5 primer and unique barcode i7 primer to amplify the library. The size distribution of the library was determined using the Agilent Tape Station 4200 (Agilent Technologies). Finally, these libraries were sequenced on the Illumina NovaSeq6000 platform, generating 150 bp paired-end reads for subsequent analysis.

### 2.8 The measurement of Ca^2+^ content and transient changes

For Ca^2+^ content assay, Fluo-4 calcium ion assay kit (Beyotime, S1061S) was used following the manufacturer's instructions by flow cytometry (Beckman Coulter, CytoFLEX). In the Ca^2+^ transient changes assay, the green fluorescence channel was selected and the duration was set to 200 s. The Ca^2+^ level in the control group was established as the baseline. Cells from other treatment groups were introduced after 50 s of running time to observe the immediate changes in calcium ion levels.

### 2.9 Microscale thermophoresis (MST)

MST determination was conducted using a Monolith NT.115 instrument (NanoTemper Technologies, Munich). First, we labeled recombinant MICU1 protein (Origene, TP300921) with NHS NT-647 dye (NanoTemper Technologies, MO-L001) and then diluted the labeled proteins with PBS to reach a final concentration of 400 nM. ACT was serially diluted 16-fold in PBS containing 10% DMSO starting from an initial concentration of 400 μM. Subsequently, we mixed the labeled MICU1 protein with ACT at a ratio of 1:1 for 10 min and then loaded it into standard capillary tubes (NanoTemper Technologies, MO-K025). Measurements were performed using 20% LED power, and KD was calculated based on the thermophoresis T-Jump results.

### 2.10 Statistical analysis

All data were independently repeated at least three times and expressed as mean ± SEM. Data comparison was performed using a One-way ANOVA among multiple groups in GraphPad Prism Software 9.0 (GraphPad, San Diego, CA). *P* ≤ 0.05 was considered as statistically significant. Additional detailed information for all involved reagents, antibodies or other experiments was provided in the Supplementary file.

## 3. Results

### 3.1 ER homeostasis and respiratory-related metabolic processes at the crux of acteoside in improving HIRI

Initially, we sought to identify critical targets underlying the abnormal changes observed in clinical data from pre- and post-liver transplant patients to determine the possible cause of HIRI. After analyzing the top 10,000 genes expression profiles of patients before and after liver transplantation in the GSE12720, GSE112713 and GSE14951, we identified 4,346 intersecting genes (**Figure 1A**). Significant alterations in ER function (protein folding, Ca^2+^ transport)-related genes [activating transcription factor 4 (*Atf4*), DNA damage inducible transcript 3 (*Ddit3,* CHOP),* inositol 1,4,5-trisphosphate receptor type 1* (*Itpr1*, IP3R), mitochondrial calcium uniporter (*Mcu*, MCU)] and glycolysis-related genes [pyruvate kinase m (*Pkm*), glyceraldehyde-3-phosphate dehydrogenase (*Gapdh*), dynamin 1 like (*Dnm1l*), lactate dehydrogenase a (*Ldha*)] were observed across all three datasets following HIRI (**Figure 1B**). We then validated the anti-HIRI effects of ACT in animal experiments and whether they were consistent with clinically relevant phenomena. Focusing on the characteristics of HIRI-induced oxidative stress and provocative inflammation, ACT significantly mitigated the elevation of aminotransferases (**Figure S1A** and **S1B**). Furthermore, in contrast to the typical liver pathological manifestations observed in the HIRI group, the ischemic area (indicated by a dotted line) in all ACT groups exhibited notable improvement (**Figure 1C**). To reveal the transcriptional landscape of ACT improving HIRI, we further performed liver RNA sequencing analysis, and genes from cluster 2 (931 genes) and cluster 3 (1,045 genes) were significantly enriched in ER function (protein folding, Ca^2+^ transport) and energy metabolism (cell respiration) process-related terms (**Figure 1D**). The bubble chart verified the top 15 target genes that were significantly enriched in cluster 2 and cluster 3. Notably, ACT significantly reversed the HIRI-induced increase in ER function-related genes [*Atf4*, *Ddit3*, ATPase sarcoplasmic/endoplasmic reticulum Ca^2+^ transporting 2 (*Atp2a2*), voltage dependent anion channel 1 (*Vdac1*, VDAC1), mitochondrial calcium uniporter regulator 1 (*Mcur1*), eukaryotic translation initiation factor 2 alpha kinase 3 (*Eif2ak3*, PERK*)*, *Itpr1*, mitochondrial calcium uptake 1 (*Micu1*, MICU1*)*, *Mcu*] and glycolysis-related genes [*Ldha*, *Pkm*, hexokinase 2 (*Hk2*), glucose-6-phosphate dehydrogenase (*G6pdx*)] (**Figure 1E**). During the analysis of liver ultrastructure, we notably observed that ACT exerted significant potential to enhance the hepatic sinusoidal microenvironment, particularly concerning the LSECs “fenestrae” (**Figure 1F**). However, the interaction patterns and target cells of the signaling network associated with Ca^2+^-centered ER dysfunction and glycolysis-dominated respiratory-related metabolic processes in sinusoidal space of HIRI and ACT-treated groups remain unclear and requires further investigation.

### 3.2 Acteoside enhances ER function before restoring mitochondrial homeostasis in LSECs damaged by hypoxia

**Figure 2:**
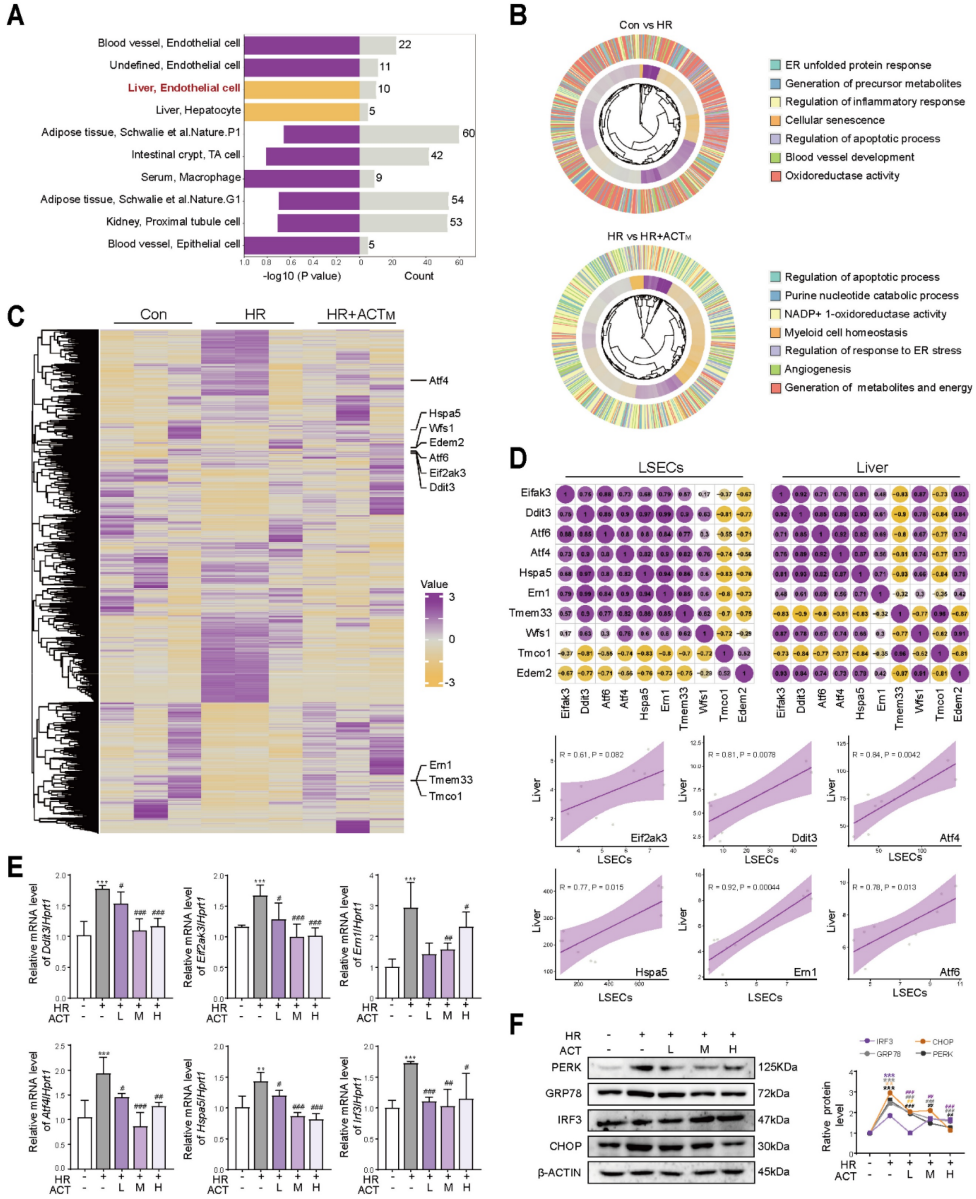
**ACT restores ER-mitochondrial homeostasis in HR-stimulated LSECs** (**A**) Association analysis of liver gene transcription and cell types (*n* = 389 cell types). (**B**) The GO analysis of representative DEGs in Con group *vs.* HR group and HR group *vs.* HR+ACT group. (**C**) Heatmap of ER-related transcripts in LSECs. (**D**) Correlation analysis of ER core genes in liver and LSECs. (**E**) The mRNA levels of *Eif2ak3*, *Ddit3*, *Hspa5*, *Atf4*, *Ern1*, *Irf3* and normalized with *Hprt1* in LSECs. (**F**) The protein levels of PERK, GRP78, IRF3, CHOP and β-ACTIN in LSECs. Data were presented as mean ± SEM (n= 3 for each group). **P < 0.01, ***P < 0.001, compared to the con group; ^#^*P* < 0.05, ^##^*P* < 0.01, ^###^*P* < 0.001, compared to the HR group by two-way ANOVA (**E** and **F**). See also **Figure S2**. ACT, acteoside; CHOP, C/EBP homologous protein; *Ddit3*, DNA damage inducible transcript 3; *Eif2ak3*, eukaryotic translation initiation factor 2 alpha kinase 3; *Ern1*, endoplasmic reticulum to nucleus signaling 1; GRP78, glucose-regulated protein 78; HR, hypoxia/re-oxygenation; Hspa5, heat shock protein family A (Hsp70) member 5; *Irf3*, interferon regulatory factor 3; LSECs, liver sinusoidal endothelial cells; PERK, protein kinase RNA-like endoplasmic reticulum kinase.

After correlating the different cell markers in 389 cell types with covarying gene modules expressed in the above groups, we indicated that the transcription of liver genes post-ACT treatment was selectively associated with LSECs programs (**Figure 2A**). Subsequently, we isolated primary LSECs, exposed to HR model for mimicking HIRI pathological state *in vitro*, followed by single cell-type RNA sequencing (**Figure S2A**). GO analysis revealed significant enrichment of genes affected by HIRI or ACT, particularly in modules related to ER function. Notably, the term “Generation of metabolites and energy” emerged as a key enriched term following ACT administration, suggesting that the positive response of ACT to LSECs energy metabolism might stem from the perturbation of ER dysfunction, further influencing the mitochondrial-dominated energy metabolism process (**Figure 2B**). The heatmap illustrated representative transcripts associated with ER functional annotation (**Figure 2C**) like *Atf4*, *Ddit3* and *Eif2ak3* involved in **Figure 2D**, upper panel. Correlation analysis demonstrated a high degree of uniformity among samples in both whole liver and single LSECs type groups, with high coupling correlations observed in the expression of core genes related to ER function [*Eif2ak3*, *Ddit3*, heat shock protein family a member 5 (*Hspa5,* GRP78), *Atf4*] (**Figure 2D**, lower panel). Consistently, ACT significantly reversed the HR-induced transcription of selected genes responsible for ER stress including *Ddit3*, *Eif2ak3*, endoplasmic reticulum to nucleus signaling 1 (*Ern1*), *Atf4*, *Hspa5*, interferon regulatory factor 3 (*Irf3*) and *Atf6* (**Figure 2E**), and translational levels of proteins including PERK, GRP78, IRF3 and CHOP that related to the above ER protein folding functions (**Figure 2F**). Concurrently, cytoskeleton associated protein 4 (CKAP4) and neurite outgrowth inhibition protein (NOGO) were utilized to visualize the ER morphology, revealing an increase in CKAP4/NOGO double-positive clusters and ER extension (more tubular ER) following HR, which was significantly ameliorated by ACT (**Figure S2B**). Collectively, our results suggest that ACT may play a critical role in mitigating HIRI damage by modulating ER function first and restoring mitochondrial homeostasis through an as-yet-undefined signaling link in LSECs.

### 3.3 Acteoside inhibits the overflow of Ca^2+^ from the ER to mitochondria and LSECs damage by influencing MICU1-regulated IP3R-VDAC1-MCU axis

**Figure 3:**
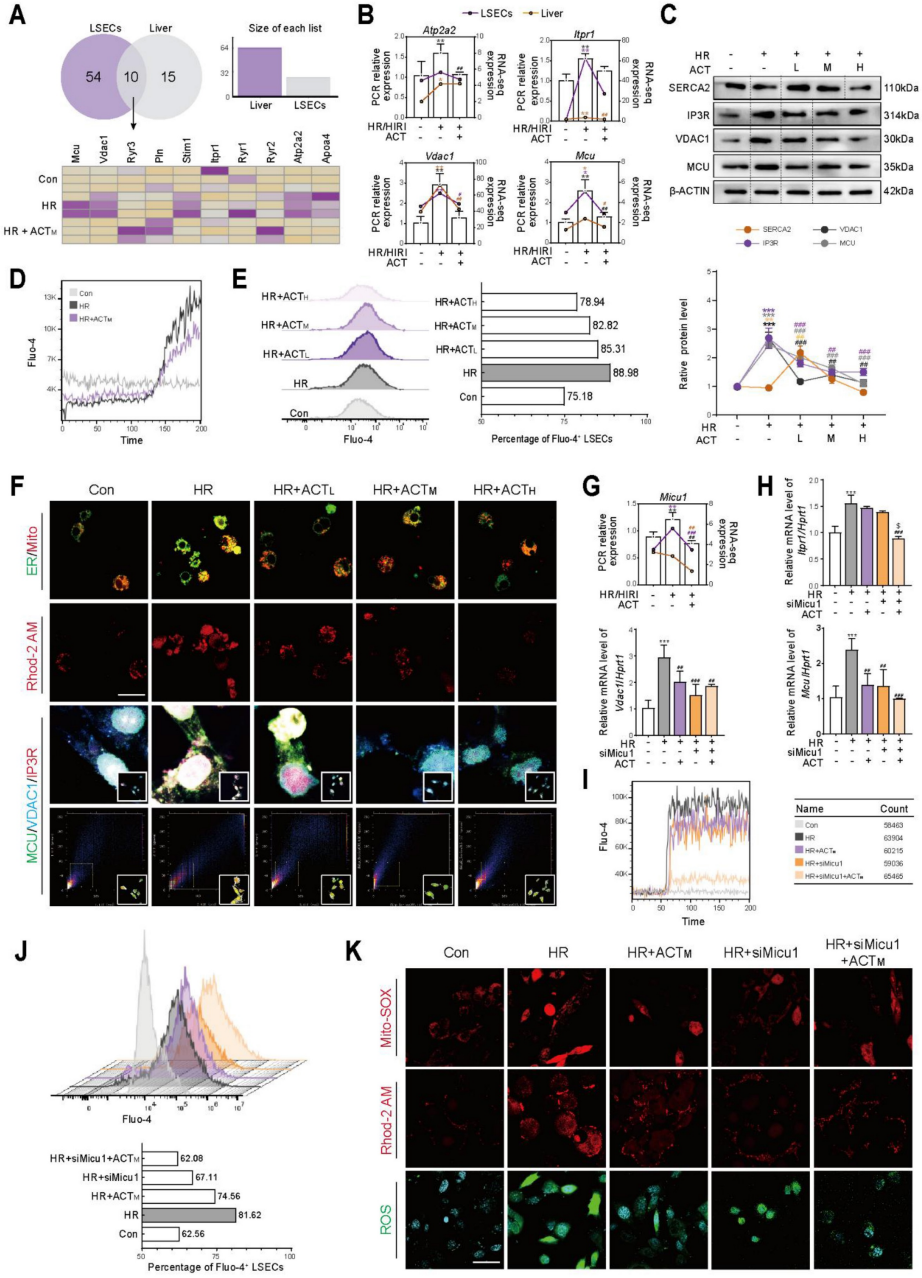
**ACT blocks the Ca^2+^ transfer from ER to mitochondrial *via* MICU1** (**A**) Venn diagram of HIRI/HR-altered genes in liver/LSECs. (**B**) The mRNA levels of *Atp2a2*, *Itpr1*, *Vdac1*, *Mcu* and normalized with *Hprt1* in liver and LSECs. (**C**) The protein levels of SERCA2, IP3R, VDAC1, MCU and β-ACTIN in LSECs. (**D**) The Ca^2+^ transient kinetics and (**E**) intracellular Ca^2+^ levels in LSECs. (**F**) Representative images of ER-mitochondria contact sites, Rhod-2 and multiplex immunofluorescence staining against IP3R, VDAC1 and MCU in LSECs, scale bar = 20 μm. (**G**) The mRNA levels of *Micu1* and normalized with *Hprt1* in liver and LSECs. (**H**) The mRNA levels of *Itpr1*, *Vdac1*, *Mcu* and normalized with *Hprt1* in LSECs treated with siMicu1 and ACT. (**I**) The Ca^2+^ oscillation kinetics and (**J**) intracellular Ca^2+^ levels in LSECs treated with siMicu1 and ACT. (**K**) Representative images of mito-SOX, Rhod-2 and ROS, scale bar = 20 μm. Data were presented as mean ± SEM (n= 3 for each group). **P < 0.01, ***P < 0.001, compared to the con group; ^#^*P* < 0.05, ^##^*P* < 0.01, ^###^*P* < 0.001, compared to the HR group; ^$^*P* < 0.05, compared to the relative HR + siMicu1 group by two-way ANOVA (**B**, **C**, **G** and **H**). See also **Figure S3**. ACT, acteoside; *Atp2a2*, ATPase sarcoplasmic/endoplasmic reticulum Ca^2+^ transporting 2; ER, endoplasmic reticulum; HR, hypoxia/re-oxygenation; IP3R, inositol 1,4,5-trisphosphate receptor; *Itpr1*, inositol 1,4,5-trisphosphate receptor type 1; *Mcu*, mitochondrial calcium uniporter; Mito, mitochondria; ROS, reactive oxygen species; SERCA2, sarcoplasmic/endoplasmic reticulum Ca^2+^-ATPase 2; *Vdac1*, voltage-dependent anion channel 1.

ER-mitochondrial contact offers a dynamic platform for physiological and biochemical communication, facilitating the exchange of Ca^2+^, ROS and lipids within cells 18. Considering the results in **Figure 2**, we focused on the changes in genes related to the transfer process of substances between ER and mitochondria in primary LSECs and preliminarily confirmed the involvement of Ca^2+^ but no other signals in ACT-induced protective effects. Subsequently, the significantly altered genes in the HIRI and HR groups from the liver/LSECs sequencing were visualized using a Venn diagram, followed by a heatmap visualization that included the ACT group (**Figure 3A**, upper panel and **Figure S3A**). As expected, the expression of Ca^2+^ transport-related genes (*Mcu*, *Vdac1*,* Itpr1* and* Atp2a2*) was partially downregulated after ACT administration (**Figure 3A**, lower panel), which was confirmed by molecular biology experiments both in livers and LSECs (**Figure 3B**, **3C** and **Figure S3B**). Then, we measured the amplitude of the Ca^2+^ transient response (**Figure 3D** and **Figure S3C**) and intracellular content (**Figure 3E**) to monitor the spontaneous Ca^2+^ transient behavior of LSECs and assess the intracellular Ca^2+^ status under HR or HR + ACT intervention. Notably, ACT intervention significantly restored Ca^2+^ homeostasis in LSECs following HR. Given the expansion of the ER structure in HR (**Figure S2B**), we hypothesized that the active edge sites exposed on the ER may enhance contact with mitochondria, thereby facilitating percolation pathways for the delivery of excess Ca^2+^ between these organelles. As shown in **Figure 3F** upper panel and **Figure S3D** and S3E, ACT-reversible increases in both ER-mitochondria co-localization and Rhod-2M labeled mitochondrial Ca^2+^ were observed after HR stimulation. ACT significantly reduced the signal coincidence of the IP3R (located in the ER)-VDAC1 [(located in the outer mitochondrial membrane (OMM)]-MCU [(located in the inner mitochondrial membrane (IMM)] complex at the ER-mitochondria contact sites (**Figure 3F** lower part, **Figure S3F** and **S3G**).

MICU1, referred to as the “switch protein” of MCU, is capable of sensing fluctuations in cytoplasmic Ca^2+^ concentration and regulating the unidirectional transport of Ca^2+^ into the mitochondria. Interestingly, unlike the moderate impact on MCU, we observed a significant increase in the mRNA expression of *Micu1* following HIRI/HR, which was significantly decreased by ACT administration. This change was highly consistent with the sequencing data obtained from liver or LSECs (**Figure 3G** and **Figure S3H**). This raises the question of whether MICU1 is a key target to regulate IP3R-VDAC1-MCU complex and the behavior of Ca^2+^ import from ER into mitochondria and subsequent mitochondrial homeostasis. To investigate this, we utilized siMicu1 to transfect LSECs (**Figure S3I**) and discovered that ACT synergistically inhibited the mRNA levels of the IP3R-VDAC1-MCU complex in conjunction with siMicu1 (**Figure 3H**), thereby enhancing the blockade of Ca^2+^ entry into the mitochondria. The depiction of the kinetic curves of Ca^2+^ oscillations (**Figure 3I** and **Figure S3J**) and intracellular Ca^2+^ levels (**Figure 3J** and **Figure S3K**) after the indicated treatments in LSECs further corroborated this phenomenon. Additionally, a combined analysis of MitoSOX, Rhod-2 AM and intracellular ROS levels demonstrated that ACT, in conjunction with siMicu1, alleviated superoxide accumulation caused by HR-induced mitochondrial Ca^2+^ overload stress (**Figure 3K** and **Figure S3L**). These findings suggest that ACT may inhibit Ca^2+^ transfer from ER to mitochondria *via* disrupting the dynamic scaffolds of the IP3R-VDAC1-MCU complex mediated by MICU1, thereby preventing mitochondrial Ca^2+^ overload and subsequent superoxide accumulation in LSECs.

### 3.4 Acteoside inhibits Ca^2+^-stimulated glycolysis by directly interacting with and blocking MICU1

**Figure 4:**
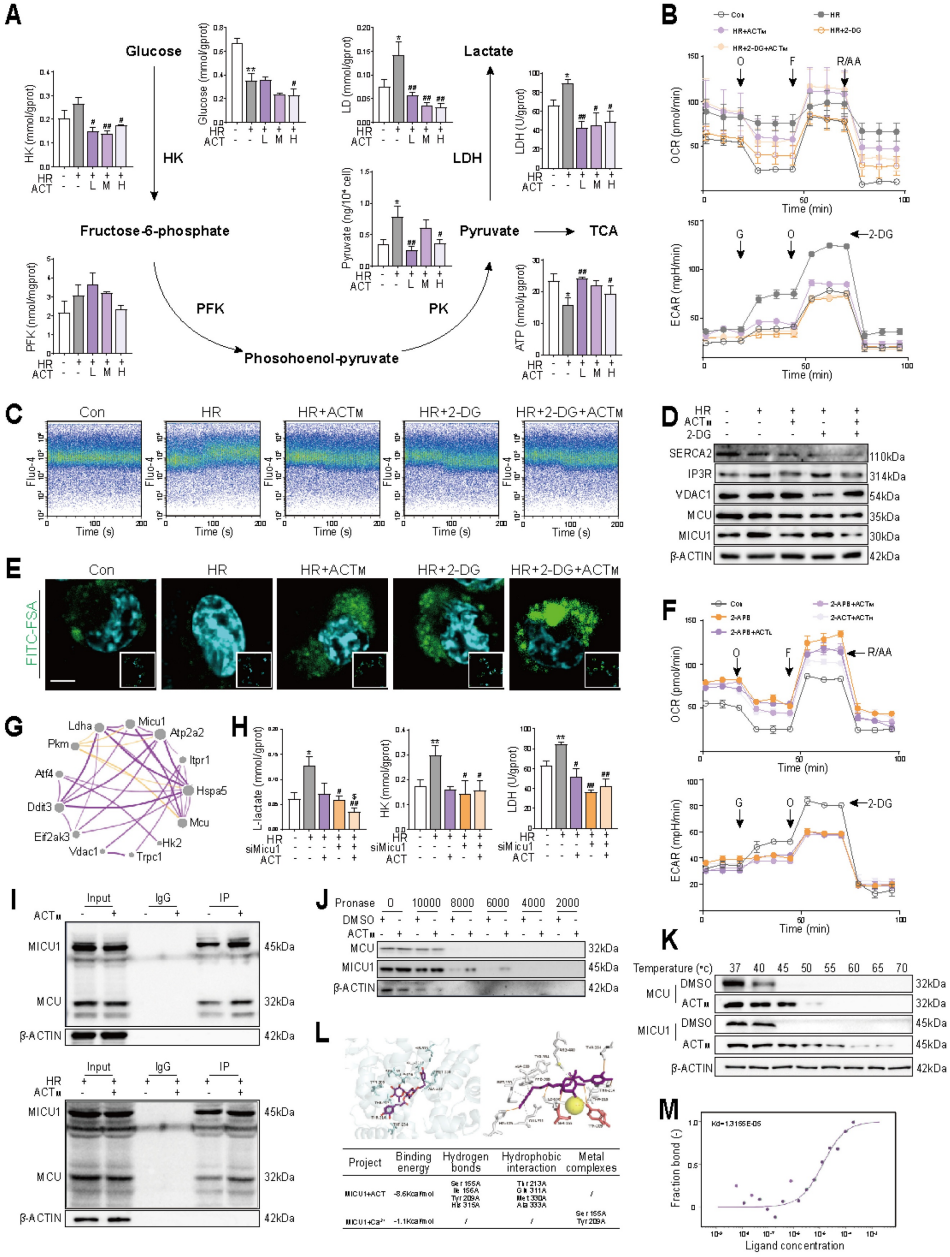
** ACT suppresses Ca^2+^-stimulated glycolysis by directly binding to MICU1** (**A**) Enzyme activities (HK, FPK, PK and LDH), lactate and ATP content involved in glycolysis process of LSECs. (**B**) OCR and ECAR assays in LSECs treated with 2-DG and ACT. (**C**) Ca^2+^ levels in LSECs treated with 2-DG and ACT. (**D**) The protein levels of SERCA2, IP3R, VDAC1, MCU, MICU1 and β-ACTIN in LSECs. (**E**) Representative images of FITC-FSA staining, scale bar = 20 μm. (**F**) OCR and ECAR assays in LSECs treated with 2-APB and ACT. (**G**) Correlation network of *Micu1* and glycolysis-Ca^2+^ transport genes. (**H**) The content of L-lactate and the activity of HK and LDH in LSECs treated with siMicu1 and ACT. (**I**) Co-IP of MICU1 with MCU in LSECs treated with ACT. (**J**) The DARTS results of MCU and MICU1 in LSECs under different concentration of pronases. (**K**) The CETSA results of MCU and MICU1 in LSECs under different temperature. (**L**) Molecular docking of MICU1 + ACT and MICU1 + ACT + Ca^2+^. (**M**) MST affinity measurements of MICU1 and ACT. Data were presented as mean ± SEM (n= 3 for each group). *P < 0.05, **P < 0.01, compared to the con group; ^#^*P* < 0.05, ^##^*P* < 0.01, compared to the HR group; ^$^*P* < 0.05, compared to the relative HR + siMicu1 group by two-way ANOVA (**A** and **H**). See also **Figure S4**. 2-APB, 2-aminoethyl diphenylborinate; 2-DG, 2-deoxy-D-glucose; ACT, acteoside; ECAR, extracellular acidification rate; F, [FCCP, (carbonyl cyanide-4-(trifluoromethoxy)phenylhydrazone)]; FITC, fluorescein isothiocyanate; FSA, formaldehyde-treated serum albumin; HK, hexokinase; HR, hypoxia/re-oxygenation; LDH, lactate dehydrogenase; MCU, mitochondrial calcium uniporter; MICU1, mitochondrial calcium uptake 1; O, oligomycin; OCR, oxygen consumption rate; PFK, phosphofructokinase; PK, pyruvate kinase; R/AA, rotenone/antimycin A; SERCA2, sarco/endoplasmic reticulum Ca^2+^-ATPase 2; IP3R, inositol 1,4,5-trisphosphate receptor; VDAC1, voltage-dependent anion channel 1; TCA, tricarboxylic acid cycle.

Research reports indicate that unfolded protein responses in the ER can release Ca^2+^, thereby influencing the glycolysis process 19, 20, which was also supported by our results shown in **Figure 2B**. Interestingly, a biphasic volcano plot revealed significant differences in glycolysis-related genes (*Gapdh*, *G6pdx*,* Ldha*, *Hk2*, *Pkm*) between the HR and HR + ACT groups (**Figure S4A**). Furthermore, the expression changes of glycolysis-genes in LSECs and liver tissues demonstrated a strict correlation (**Figure S4B** and **S4C**). Consistently, the activities of key enzyme [hexokinase (HK), phosphofructokinase (PFK) and lactate dehydrogenase (LDH)] activities, as well as mRNA and protein levels of glycolytic metabolites and their corresponding canonical glycolytic enzymes, were significantly reduced after ACT administration (**Figure 4A**, **Figure S4D** and **S4E**). Concurrently, ACT alleviated the accumulation of lactic acid and the insufficient supply of ATP caused by HIRI (**Figure 4A**). The results of oxygen consumption rate (OCR) and extracellular acidification rate (ECAR) further confirmed that glycolytic reserves were significantly reduced in the HR+ACT group. However, no additional inhibition was observed upon the addition of the glycolytic inhibitor 2-deoxyglucose (2-DG). This suggested that the mechanism by which ACT inhibits HR-induced glycolysis is distinct from that of 2-DG, potentially mediated through the Ca^2+^ signaling pathway (**Figure 4B** and **Figure S4F**). As expected, ACT exhibited a role similar to that of 2-DG, significantly reducing Ca^2+^ levels (**Figure 4C**, **4D**, **Figure S4G** and **S4H**) and restoring normal function of LSECs after HR (**Figure 4E**). To further elucidate the connection between Ca^2+^ signaling and glycolysis process, we administered 2-APB (a Ca^2+^ influx inhibitor) to LSECs. The results indicated that, in comparison to 2-APB alone, the combination of 2-APB and ACT did not result in a further reduction of glycolytic reserve. This suggested that ACT may inhibit overactivated glycolysis *via* the Ca^2+^ amplification pathway, rather than through an independent mechanism (**Figure 4F**). This suggested that HR-activated glycolysis may be regulated by intracellular aberrant Ca^2+^ signals and also reversed by ACT.

In addition to regulating MCU target, MICU1 is also able to function as a metabolic switch, mediating glycolytic processes driven by Ca^2+^ influx into mitochondria 21. Under HR conditions, we assessed the correlation between MICU1 and key genes involved in glycolysis (*Hk2, Ldha, Pkm*) and Ca^2+^ transport (*Mcu, Vdac1, Eif2ak3, Itpr1*), trying to uncover the potential relationship among MICU1, alterations in ER functionality and glycolytic process (**Figure 4G**). As shown in **Figure 4H** and **Figure S4I**, ACT synergized with siMicu1 to inhibit the activities of key glycolytic enzymes, especially for LDH and increase ATP content, accompanied by the decreased accumulation of L-lactate, but not D-lactate levels in LSECs. Meanwhile, ACT primarily functioned in conjunction with siMicu1 to reduce glycolytic levels (**Figure S4J**) and significantly inhibit the HR-induced increase in Ca^2+^ levels in LSECs (**Figure S3I**). After observing that ACT can affect the IP3R-VDAC1-MCU complex and subsequent processes through MICU1, we sought to further elucidate the interaction mode between ACT and MICU1, MCU. It is heartening to note that ACT dramatically enhanced the formation and binding of MICU1-MCU complexes under either normal (**Figure 4I**, upper part) or HR (**Figure 4I**, lower part) conditions. The consistent DARTS and CETSA results directly indicated that ACT exhibited a stronger binding affinity for MICU1 compared to MCU in LSECs (**Figure 4J** and **4K**). Consequently, we conducted molecular docking simulations to examine the interaction between ACT, Ca^2+^ and MICU1. Our findings revealed that ACT tightly bound to MICU1 protein (**Figure S4K**) and appeared to compete with Ca^2+^ for the active site of MICU1 (**Figure 4L**), potentially inhibiting the transport of Ca^2+^ by the MCU-MICU1 complex. To further substantiate the interaction between ACT and MICU1 and to quantify the binding affinity, we utilized MST and confirmed positive binding of ACT to MICU1 (**Figure 4M**). Overall, these results corroborated that the robust interaction between ACT and MICU1 can disrupt the interaction between MICU1 and Ca^2+^, inhibit glycolysis induced by MICU1-Ca^2+^ perturbation and lower intracellular lactate levels following HR.

### 3.5 Acteoside inhibits *Micu1* transcription by suppressing H3K18 lactylation in LSECs

**Figure 5:**
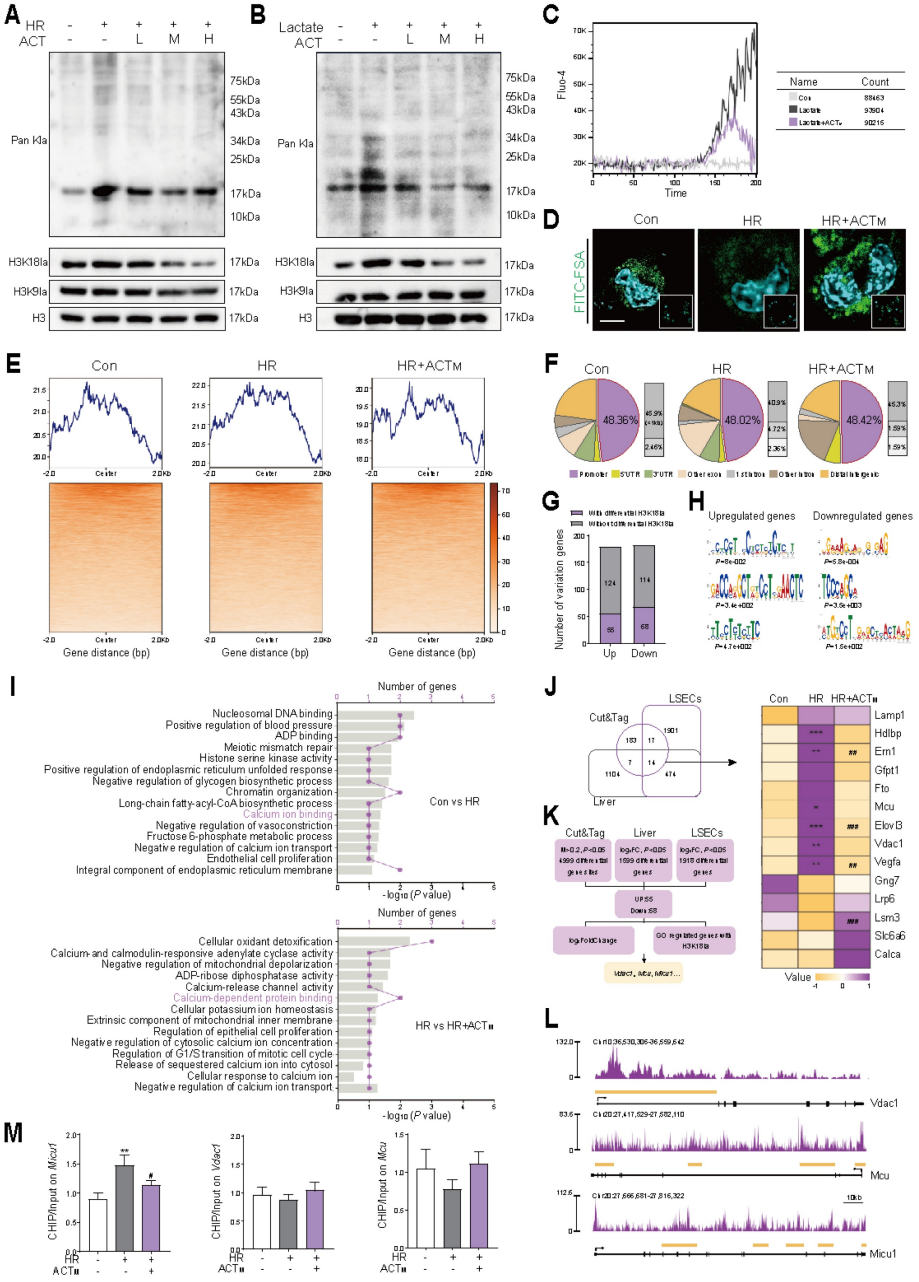
** ACT attenuates H3K18la-driven Ca^2+^ dysregulation through MICU1 suppression in HR-induced LSECs** Global lactylation, H3K18la and H3K9la levels of LSECs (**A**) in HR or (**B**) lactate model. (**C**) Ca^2+^ transient kinetics in LSECs. (**D**) Representative images of FITC-FSA staining in LSECs, scale bar = 20 μm. (**E**) Heatmaps for H3K18la binding peaks in LSECs from Con, HR, HR+ACT. All quality metrics were excellent, including Q30 (90%), valid bases (91-92%) and GC content (46%). (**F**) Genomic distribution of H3K18la peaks in the promoter region. (**G**) The number of up-regulated and downregulated genes with differential H3K18la modification. (**H**) The top 3 enriched *de novo* motifs of the upregulated and downregulated genes with differential H3K18la modification. (**I**) GO terms linked to Ca^2+^ transport pathways in regulated H3K18la-modified genes. (**J**) Venn diagram analysis of the overlap of CUT&TAG, liver transcriptomes and LSECs transcriptomes. (**K**) Flow diagram about the downstream targets of H3K18la. (**L**) H3K18la peaks in the promoter regions of *Mcu*, *Vdac1* and *Micu1*. (**M**) ChIP-qPCR analytics of H3K18la occupancy. Data were presented as mean ± SEM (n= 3 for each group). *P < 0.05, **P < 0.01, ***P < 0.001, compared to the con group; ^#^*P <* 0.05, ^##^*P <* 0.01, ^###^*P <* 0.001, compared to the HR group by two-way ANOVA (**J** and **M**). See also **Figure S5**. ACT, acteoside; CHIP, chromatin immunoprecipitation; FITC, fluorescein isothiocyanate; FSA, formaldehyde-treated serum albumin; H3K18la, histone H3 lysine 18 lactylation; HR, hypoxia/re-oxygenation; MCU, mitochondrial calcium uniporter; MICU1, mitochondrial calcium uptake 1; Pan Kla, pan-lysine lactylation; VDAC1, voltage-dependent anion channel 1.

Considering the established association between glycolysis and histone lactylation 6, together with our existing results (**Figure 4**), we inquired whether ACT could influence these processes to ameliorate HR injury, and established models for HR and lactate accumulation. As illustrated in **Figure 5A**, **5B** and **Figure S5A**, ACT significantly reduced the levels of global lactylation and H3K18la induced by HR or excessive lactate in a dose-dependent manner. Concurrently, the accumulation of lactate, a by-product of glycolysis, was associated with an increase in intracellular Ca^2+^ (**Figure 5C**, **Figure S5B**) and induced Ca^2+^ channel protein expression on the ER-mitochondrial axis (**Figure S5C**). This Ca^2+^ increase further enhanced intracellular glycolysis (**Figure S5D**) and induced phagocytic dysfunction in LSECs (**Figure 5D** and **Figure S5E**). Notably, these effects were effectively reversed through chemical intervention with ACT. To screen candidate target genes regulated by histone lactylation in HR, CUT&Tag was performed with ChIP-grade H3K18la antibody and IgG in LSECs (**Figure 5E**). **Figure** 5F showed that H3K18la was enriched in promoter regions of genes (especially the regions < 1 kb) in all groups. To identify H3K18la-target genes in LSECs, we classified genes into 4 categories according to their expression and H3K18la modification: upregulated genes with or without differential H3K18la, and downregulated genes with or without differential H3K18la (**Figure 5G** and **Figure S5F**). Through a *de novo* motif search among the 2 categories of H3K18la-modified genes, we found that upregulated and downregulated genes with H3K18la exhibited distinct enriched DNA sequences (**Figure 5H**). GO term analysis revealed that genes with increased H3K18la modification during HR were actively involved in the regulation of Ca^2+^ transport, indicating the pivotal role of H3K18la in Ca^2+^ transport regulation (**Figure 5I**). We subsequently integrated CUT&Tag data with LSECs transcriptome sequencing and whole liver transcriptome sequencing results, and *Vdac1*, *Mcu* and *Micu1* were selected as candidate genes (**Figure 5J** and **5K**). The signals of enriched H3K18la in the promoter regions of *Vdac1*, *Mcu* and *Micu1* were observed in **Figure 5L** and **Figure S5G**. The enrichment of H3K18la at the promoter regions of *Vdac1* and *Micu1* was less prominent after ACT intervention. Subsequently, ChIP-qPCR analysis indicated that ACT effectively reduced the enrichment of H3K18la in the promoter region of *Micu1*, but not in *Vdac1* or *Mcu* (**Figure 5M**). Notably, these HR-induced pathological alterations and their reversal by ACT were also validated in human LSECs, showing consistent attenuation of ER stress, Ca^2+^ dysregulation, glycolytic lactate production and H3K18la modification (**Figure S9**).

### 3.6 Acteoside synergizes with lactate inhibitors to maintain Ca^2+^ homeostasis through negative regulation of H3K18la-Micu1 signaling

**Figure 6:**
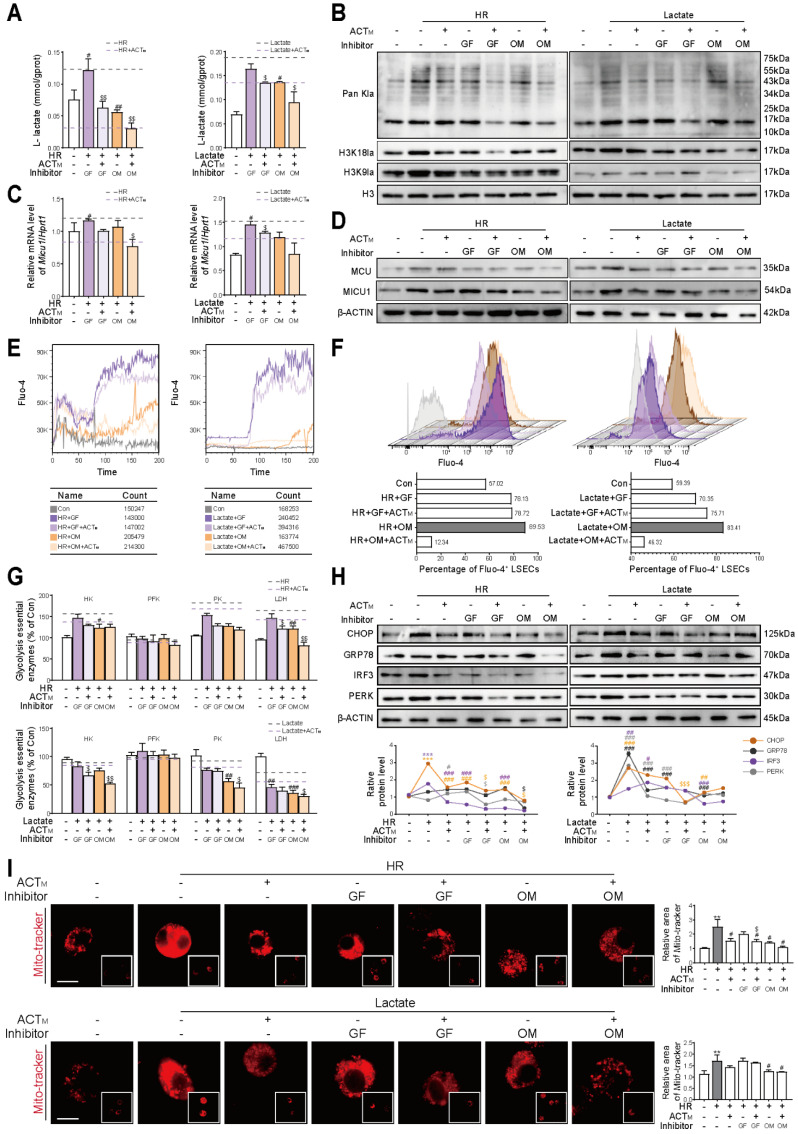
** ACT and lactate inhibitors synergistically inhibit H3K18la to maintain MICU1-regulated Ca^2+^ homeostasis** (**A**) L-lactate content, (**B**) global histone lactylation, H3K18la and H3K9la levels of LSECs treated with galloflavin or oxamate in exogenous HR or lactate models. (**C**) The mRNA levels of *Micu1* in LSECs treated with galloflavin or oxamate in exogenous HR or lactate models. (**D**) The protein levels of MCU and MICU1 in LSECs treated with galloflavin or oxamate in exogenous HR or lactate models. (**E**) Ca^2+^ transient amplitude and (**F**) intracellular Ca^2+^ content of LSECs in exogenous HR or lactate models. (**G**) Enzyme activities and protein levels of key glycolytic regulators (HK, PFK, PK and LDH) of LSECs. (**H**) The protein levels of CHOP, GRP78, IRF3 and PERK and β-ACTIN in LSECs. (**I**) Representative images of mito-tracker staining under different conditions, scale bar = 20 μm. Data were presented as mean ± SEM (n= 3 for each group). ***P < 0.001, compared to the con group; ^#^*P* < 0.05, ^##^*P* < 0.01, ^###^*P* < 0.001, compared to the relative model (HR or lactate) groups; ^$^*P* < 0.05, ^$$^*P* < 0.01, ^$$$^*P* < 0.001compared to the relative model (HR or lactate) + inhibitor (GF or OM) groups by two-way ANOVA (**A**, **C**, **G**, **H** and **I**). See also **Figure S6**. ACT, acteoside; CHOP, C/EBP homologous protein; GF, galloflavin; GRP78, glucose-regulated protein 78; HK, hexokinase; H3K18la, histone H3 lysine 18 lactylation; HR, hypoxia/re-oxygenation; IRF3, interferon regulatory factor 3; LDH, lactate dehydrogenase; MCU, mitochondrial calcium uniporter; MICU1, mitochondrial calcium uptake 1; Mito, mitochondria; OM, oxamate; Pan Kla, pan-lysine lactylation; PFK, phosphofructokinase; PERK, protein kinase RNA-like endoplasmic reticulum kinase; PK, pyruvate kinase; VDAC1, voltage-dependent anion channel 1.

To further establish the connection between histone lactylation and Ca^2+^-stimulated glycolysis, we determined the schemes of consuming intracellular lactate with galloflavin (targeting to inhibit LDHA and LDHB, 10 μM) or oxamate (only targeting to inhibit LDHA, 5 mM) according to proliferation status of LSECs and their lactate content (**Figure S6A**). The results indicated that following the addition of exogenous lactate or HR stimulation, ACT synergistically interacted with galloflavin or oxamate and significantly inhibited the L-lactate level in LSECs. Notably, oxamate in combination with ACT achieved a more pronounced inhibitory effect on L-lactate levels than galloflavin (**Figure 6A**). Although the cooperation between ACT and galloflavin or oxamate significantly inhibited the global lactylation of LSECs under conditions of lactate accumulation or HR stimulation, consistent with **Figure 5**, this ACT-targeted lactylation also specifically occurred at H3K18 site (**Figure 6B** and **Figure S6B**). Based on the results of **Figure 3** and **Figure 5**, we speculated that *Micu1* was regulated by H3K18la and thus played a crucial role in regulating intracellular Ca^2+^ homeostasis. Following stimulation with exogenous lactate or HR, the combination of ACT with galloflavin or oxamate resulted in a more pronounced inhibition of MICU1 mRNA and protein levels compared to MCU. (**Figure 6C**, **6D** and **Figure S6C**, **S6D**). The assessment of Ca^2+^ transient response amplitude (**Figure 6E** and **Figure S6E**) and intracellular Ca^2+^ content (**Figure 6F** and **Figure S6F**) indicated that the synergy of ACT with galloflavin or particularly oxamate, mitigated intracellular Ca^2+^ dysregulation induced by HR or lactate accumulation. Regarding the Ca^2+^-stimulated glycolytic process, we observed that ACT inhibited the protein expression and enzyme activity of key glycolytic enzymes, particularly HK, pyruvate kinase (PK) and LDH with the help of galloflavin or oxamate, while exerting minimal impact on PFK expression and enzyme activity (**Figure 6G** and **Figure S6G**). Additionally, given the close relationship between fluctuations in Ca^2+^ levels and the ER-mitochondrial axis, we assessed ER stress-related proteins and monitored mitochondrial status. ACT also synergistically reduced the expression of ER stress-related proteins such as CHOP, GRP78, IRF3 and PERK (**Figure 6H**), and improved mitochondrial fragmentation (**Figure 6I**), maintaining the normal functions of ER and mitochondria in cooperation with galloflavin or oxamate. These results indicated that accumulated lactate facilitated the H3K18la modification of MICU1, thereby enhancing its ability to facilitate Ca^2+^ delivery through the ER-mitochondrial axis and further promoted glycolysis, all of which were remarkably inhibited by ACT.

### 3.7 The anti-HIRI properties of acteoside can be partially improved with the specific inhibition of *Micu1* in livers

**Figure 7:**
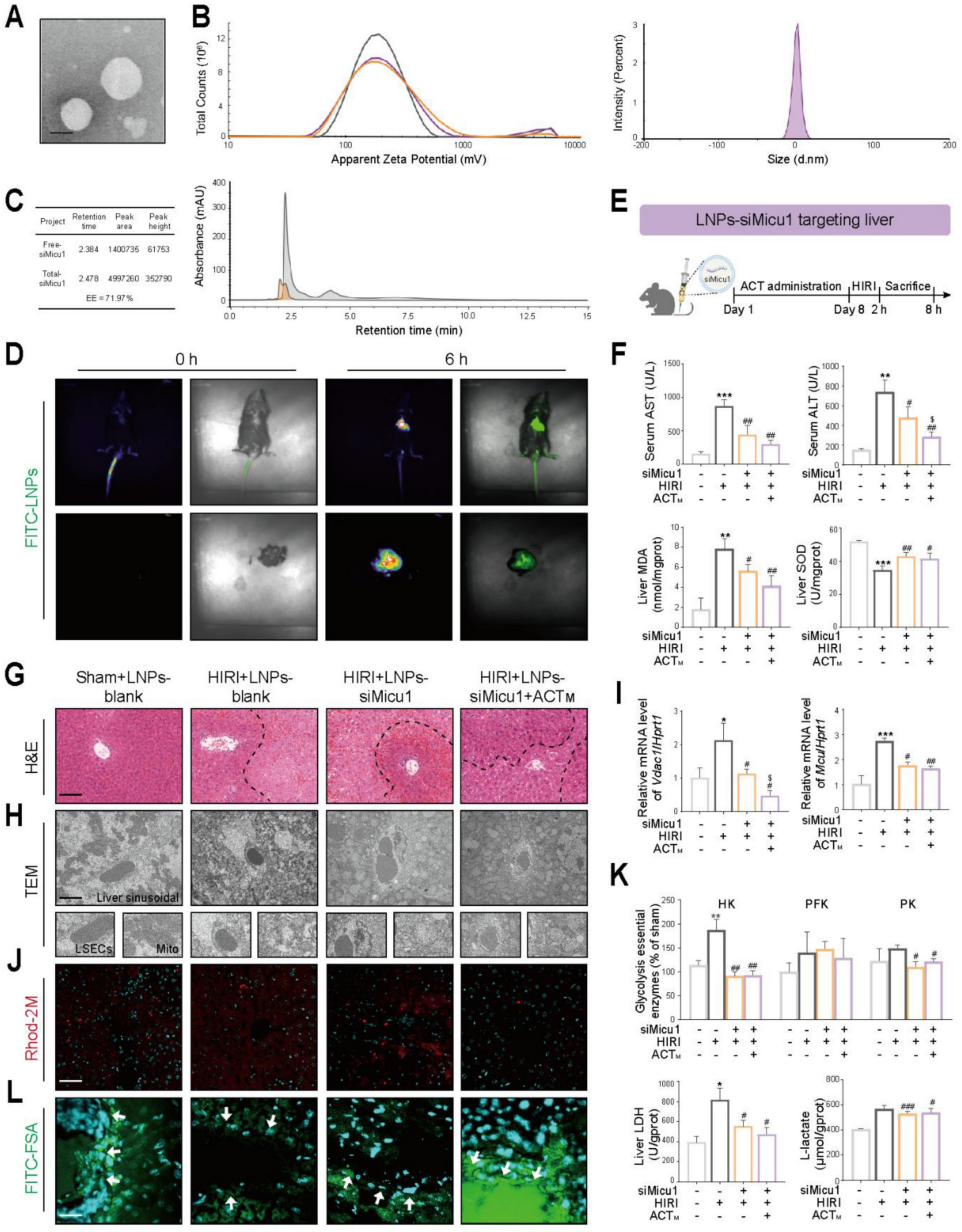
** Co-administration of ACT and siMicu1-LNPs shows more prominent improvement of mitochondrial Ca^2+^ homeostasis and HIRI *in vivo*** (**A**) TEM morphology of siMicu1-LNPs, scale bar = 200 nm. (**B**) Hydrodynamic diameter and zeta potential of siMicu1-LNPs. (**C**) Encapsulation efficiency of siMicu1-LNPs. (**D**) Biodistribution of siMicu1-LNPs* in vivo*. (**E**) Schematic diagram of animal experiments. ACT (50 mg/kg, i.p.) was administered for 7 consecutive days prior to HIRI surgery, and siMicu1-LNPs (5 μl/g, i.v.) were administered 2 h prior to HIRI surgery. (**F**) The levels of serum AST, ALT contents and hepatic MDA, SOD activities. (**G**) Representative H&E staining of liver, scale bar = 50 μm. (**H**) TEM analysis of LSECs, scale bar = 2 μm. (**I**) The mRNA levels of *Vdac1*, *Mcu* and normalized with *Hprt1* in livers. (**J**) Representative images of Rhod-2 in liver, scale bar = 50 μm. (**K**) Glycolytic enzyme activities (HK, PFK, PK and LDH) and L-lactate content in liver. (**L**) Representative images of FITC-FSA staining in liver, scale bar = 50 μm. Data were presented as mean ± SEM (n= 6 for mice). *P < 0.05, **P < 0.01, ***P < 0.001, compared to the Sham group; ^#^*P* < 0.05, ^##^*P* < 0.01, ^###^*P* < 0.001, compared to the HIRI group; ^$^*P* < 0.05, compared to the HIRI + LNPs-siMicu1 groups by two-way ANOVA (**F**, **I** and **K**). See also **Figure S7**. ACT, acteoside; CHOP, C/EBP homologous protein; GF, galloflavin; GRP78, glucose-regulated protein 78; H3, histone H3; H3K18la, histone H3 lysine 18 lactylation; H3K9la, histone H3 lysine 9 lactylation; HK, hexokinase; HR, hypoxia/re-oxygenation; IRF3, interferon regulatory factor 3; LDH, lactate dehydrogenase; MCU, mitochondrial calcium uniporter; MICU1, mitochondrial calcium uptake 1; Mito, mitochondria; OM, oxamate; Pan Kla, pan-lysine lactylation; PERK, protein kinase RNA-like endoplasmic reticulum kinase; PFK, phosphofructokinase; PK, pyruvate kinase; VDAC1, voltage-dependent anion channel 1.

To verify whether the inhibition of *Micu1* improves LSECs' function by maintaining Ca^2+^ homeostasis and inhibiting glycolysis *in vivo*, we constructed siMicu1-LNPs associated with ApoE for targeted delivery to the liver, and evaluated their quality and biological performance before injection. Following the preparation of liposomes by thin-film hydration method, TEM revealed the presence of irregular spherical nanoparticles with electron-dense core structures (**Figure 7A**). Dynamic light scattering (DLS) and Zeta-potential analysis showed that the particle size of siMicu1-LNPs was 80.03 ± 1.19 nm and the surface potential was 10.12 mV (**Figure 7B**). The encapsulation efficiency (EE%) determined by UPLC was 71.97% (**Figure 7C**). In addition, to determine the biodistribution of the siMicu1-LNPs formulation following tail vein injection, we performed *in vivo* fluorescence imaging analysis using FITC-labeled siMicu1-LNPs. Robust photon fluxes were readily detected in the abdomen at 6 h post-injection, and further *ex vivo* imaging of the heart, liver, spleen, lung and kidney clearly demonstrated that liver was the primary target organ (**Figure 7D** and **Figure S7A**). Subsequently, we intravenously injected mice with blank-LNPs or siMicu1-LNPs into mice *via* the caudal vein before HIRI surgery and ACT administration (**Figure 7E** and **Figure S7B**). Compared with the HIRI+LNPs-blank group, there was no significant change in body weight but a remarkable decrease trend of spleen coefficient in the HIRI+LNPs-siMicu1 group and HIRI + LNPs-siMicu1 + ACT group (**Figure S7C**). The increased levels of plasma AST and ALT, along with elevated MDA and reduced SOD activities induced by HIRI, were largely improved in the HIRI + LNPs-siMicu1 group, more significantly in the HIRI + LNPs-siMicu1 + ACT group (**Figure 7F**). The areas of liver sinus congestion and inflammatory cell infiltration in the HIRI + LNPs-blank group were significantly increased, but the fundamental structure of liver lobules was significantly restored in HIRI + LNPs-siMicu1 and HIRI + LNPs-siMicu1 + ACT groups (**Figure 7G**). TEM results indicated that ACT synergized with siMicu1-LNPs to ameliorate mitochondrial cristae fragmentation, Ca^2+^ accumulation in mitochondria and the “defenestration” phenotype of LSECs induced by HIRI, thereby protecting the sinusoidal microenvironment (**Figure 7H**). Consistent with *in vitro* experiments, ACT and siMicu1 synergistically inhibited the expression of genes and proteins related to Ca^2+^ transfer in the ER-mitochondrial axis (**Figure 7I** and **Figure S7D**), alleviated mitochondrial Ca^2+^ accumulation (**Figure 7J** and **Figure S7E**), and reversed the increased expression and activity of glycolysis-related enzymes induced by HIRI (**Figure 7K** and **Figure S7F**), thereby protecting the function of LSECs (**Figure 7L** and **Figure S7G**).

### 3.8 MICU1 overexpression in LSECs or the hydroxyl structures at C_26_/C_27_/C_40_/C_41_ sites determines the protective effects of acteoside on Ca^2+^ homeostasis and glycolytic flux

**Figure 8:**
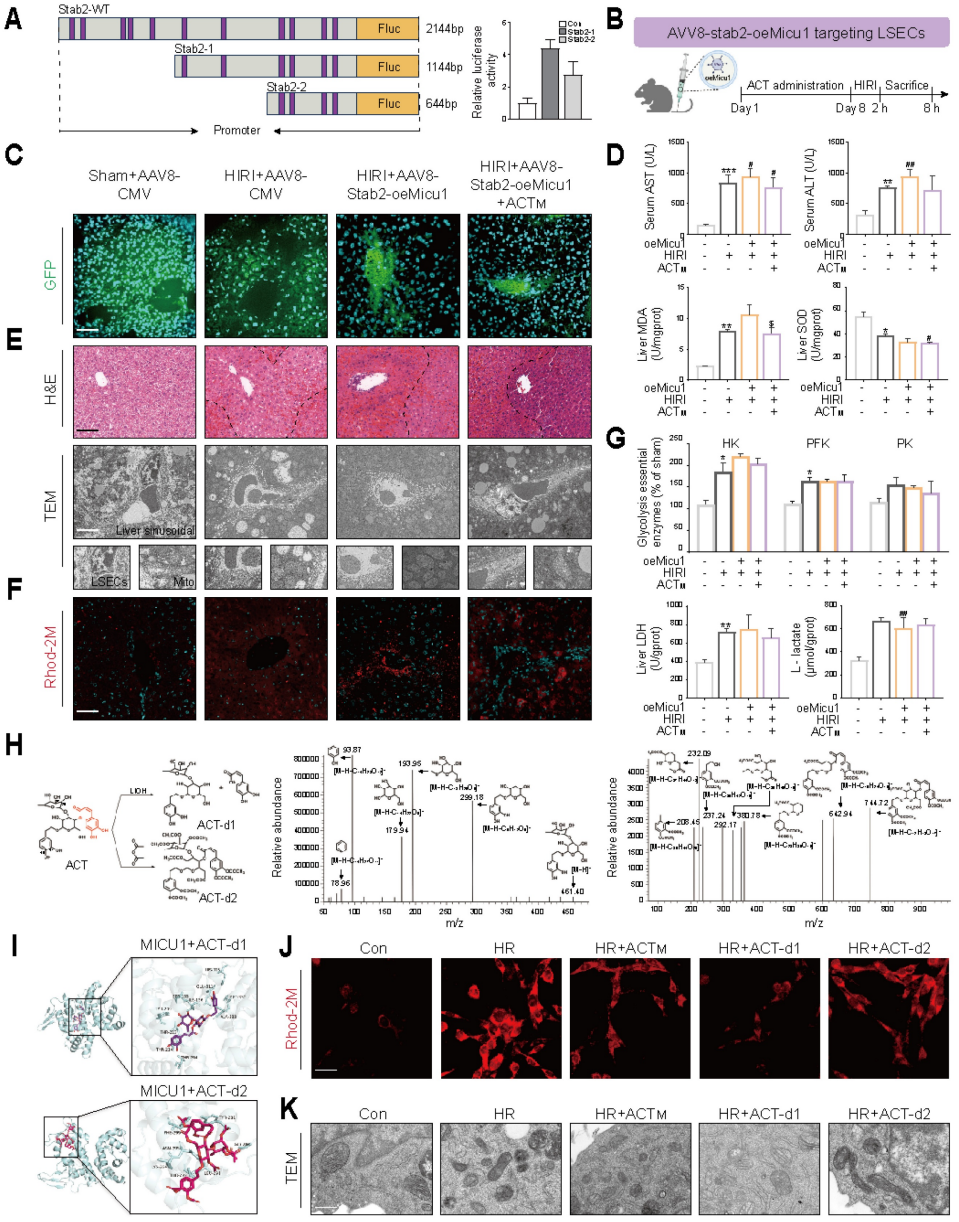
** LSECs-specific *Micu1* overexpression blocks the anti-HIRI properties of ACT in mice, which is strongly related with its specific chemical structure** (**A**) Dual-luciferase activity of truncated *Stab2* promoter in LSECs. (**B**) Schematic diagram of animal experiments. After ACT (50 mg/kg, i.p.) pretreated for 7 consecutive days, AAV8-stab2-oeMicu1 (5 μl/g, i.v.) was injected for 2 weeks prior to HIRI surgery. (**C**) Representative photofluorogram of AAV8 system in mouse livers. (**D**) The levels of serum AST, ALT contents, and hepatic MDA and SOD activities. (**E**) Representative H&E staining and TEM analysis of liver. (**F**) Representative images of Rhod-2 in liver, scale bar = 50 μm. (**G**) Glycolytic enzyme activities (HK, PFK, PK and LDH) and L-lactate content in liver. (**H**) Structural modifications of ACT derivatives and MS validation. (**I**) Molecular docking of MICU1 with ACT derivatives. (**J**) Representative images of Rhod-2 in LSECs treated with ACT derivatives. (**K**) TEM analysis in LSECs treated with ACT derivatives. Data were presented as mean ± SEM (n= 6 for mice). *P < 0.05, **P < 0.01, ***P < 0.001, compared to the Sham group; ^#^*P* < 0.05, ^##^*P* < 0.01, compared to the Sham group; ^$^*P* < 0.05, compared to the HIRI + AAV8-stab2-oeMicu1 groups by two-way ANOVA (**D** and **G**). See also **Figure S8**. ACT, acteoside; ALT, alanine aminotransferase; AST, aspartate aminotransferase; CMV, cytomegalovirus; GFP, green fluorescent protein; H&E, hematoxylin and eosin; HK, hexokinase; HR, hypoxia/re-oxygenation; LDH, lactate dehydrogenase; MDA, malondialdehyde; PFK, phosphofructokinase; PK, pyruvate kinase; SOD, superoxide dismutase; Stab2, stabilin-2; TEM, transmission electron microscopy; WT, wild-type; oeMicu1, MICU1 overexpression.

To identify the impact of MICU1 overexpression on LSECs, we screened truncated variants of the Stab2 promoter (a specific scavenger receptor expressed in LSECs) as potential candidate sequences 22. Dual-luciferase reporter assays demonstrated that a 644 bp truncated fragment (designated Stab2-2), spanning from -500 to +144 relative to the transcription start site, exhibited significantly higher activity compared to the full-length promoter and other truncations, while showing no detectable activity in hepatocytes (**Figure 8A** and **Figure S8A**). Utilizing this optimized promoter, we designed an AAV8 vector (AAV8-Stab2-oeMicu1) to specifically deliver *Micu1* to LSECs and injected it into mice 2 weeks prior to HIRI surgery with or without ACT administration (**Figure 8B** and **Figure S8B**). Following the overexpression of *Micu1* in LSECs (**Figure S8C**), we observed an increased ratio of spleen to body weight (**Figure S8D**) and a high intensity of green fluorescence in the hepatic sinusoids of mice treated with HIRI + AAV8-Stab2-oeMicu1 (**Figure 8C**). Compared to the HIRI + AAV8-CMV group, the overexpression of Micu1 resulted in more severe HIRI damage, as evidenced by significantly increased levels of serum ALT, AST and hepatic MDA levels, along with decreased SOD levels (**Figure 8D**). The HIRI+AAV8-Stab2-oeMicu1 group exhibited more pronounced hepatic sinusoidal necrosis, mitochondrial cristae fragmentation and Ca^2+^ accumulation, as well as LSECs damage (**Figure 8E**), of which pathological phenomenon was minimally alleviated by ACT. Moreover, ACT appeared to have minimal effect on reversing the alterations of Ca^2+^-related targets within the ER-mitochondrial axis (**Figure 8F** and **Figure S8E-8G**) in HIRI + AAV8-Stab2-oeMicu1 mice. Additionally, ACT didn't obviously influence the *Micu1* overexpressing-induced glycolytic flux, as evidenced by unchanged expressions and activities of HK, PK, and LDH, along with L-lactate production (**Figure 8G** and **Figure S8H**). These findings indicated that the Stab2-driven LSECs-specific *Micu1* overexpression exacerbated HIRI by promoting Ca^2+^ dyshomeostasis and metabolic reprogramming, which was no longer mitigated by ACT.

To clarify the key substituents of ACT that affect MICU1, we designed two distinct modified ACT derivatives: (1) ACT-d1 was removed the structures (highlighted in red) at the C8 sites while retaining other key hydroxyl groups responsible for binding to MICU1 and Ca^2+^. (2) ACT-d2 was removed all active hydroxyls groups (**Figure 8H**, left panel). Mass spectrometry (MS) analysis confirmed the successful structural modifications of these ACT derivatives (**Figure 8H**, middle and right panel). Structurally, ACT-d1 exhibited stronger interactions with MICU1 due to its more open molecular structure, while ACT-d2 virtually unbound compared to the unmodified ACT compound (**Figure 8I**). Functionally, ACT-d1 effectively reduced intracellular L-lactate levels and inhibited mitochondrial Ca^2+^ accumulation (**Figure 8J** and **Figure S8I**), thereby restoring the structural integrity of LSECs *in vitro* (**Figure 8K**). In contrast, ACT-d2 demonstrated negligible effects in these areas. These results validated that the modified ACT derivative (ACT-d1), which retained hydroxyls at C_26_, C_27_, C_40_ and C_41_ positions, enhanced the regulatory effects of ACT on MICU1. This structural optimization strategy provided a promising avenue for the further development of MICU1-targeted therapies to mitigate HIRI.

## 4. Discussion

HIRI arises from tightly coordinated yet highly vulnerable interactions among hepatocytes, LSECs, Kupffer cells (KCs) and infiltrating leukocytes within the hepatic sinusoid. Among these cells, LSECs form the highly discontinuous sinusoidal vascular walls that was enriched in “fenestrae”, thereby simultaneously governing sinusoidal multicellular microcirculation, leukocyte adhesion and the exchange of metabolic mediators. LSECs injury during HIRI is widely considered an early event that precipitates sinusoidal congestion, KCs activation and secondary hepatocyte death. In this study, the ultramicroscopic structural changes observed by TEM and liver gene transcription following ACT treatment were more significantly associated with LSECs than with other hepatic cell types after HIRI (**Figure 2A**). This selective targeting likely stems from the unique structural and functional roles of LSECs participate in the development of acute HIRI disease and improved LSECs-specific “fenestrate” structures facilitating ACT accumulation in the Disse space 23.

The intracellular Ca^2+^ homeostasis regulatory system primarily includes voltage-gated Ca^2+^ channels [voltage-gated calcium channels (VGCCs)], receptor-operated Ca^2+^ channels [IP3R, Ryanodine Receptor (RyR)], and mitochondrial Ca^2+^ uptake machinery, including MCU and its regulatory protein MICU1 24. Among these, MICU1 serves as a core regulator of Ca^2+^ homeostasis on the ER-mitochondrial axis. Under resting conditions (low Ca^2+^ environment), MICU1 binds to the MCU channel in an inhibitory dimeric conformation, thereby preventing mitochondrial Ca^2+^ overload. Conversely, under stress conditions (high Ca^2+^ environment), MICU1 releases its inhibition on MCU, facilitating the entry of Ca^2+^ into the mitochondria. Additionally, this bidirectional regulatory mechanism of MICU1 holds significant pathological relevance. Research has demonstrated that the overexpression of MICU1 in tumor tissues correlates with poorer overall survival rates in patients 21. Conversely, the skeletal muscle-specific knockout of MICU1 compromises mitochondrial Ca^2+^ uptake capacity, resulting in structural damage to muscle fibers 5. Interestingly, hepatocyte-specific MICU1 may exert a protective function in liver regeneration 25. These findings demonstrate the existence of tissue and cell heterogeneity of MICU1 functionality across various pathological conditions. Furthermore, as glycolysis-dependent cells, LSECs exhibit heightened susceptibility to acute ischemia-reperfusion injury, whereas their pathologically enhanced glycolysis triggers a vicious cycle of lactate accumulation and disrupted Ca^2+^ homeostasis 26. This apparent discrepancy likely arises from fundamental differences in energy utilization and Ca^2+^ handling between hepatocytes and LSECs, whereby MICU1-mediated Ca^2+^ uptake supports oxidative metabolism in hepatocytes but promotes Ca^2+^ overload and injury in glycolysis-dependent LSECs under acute HIRI. ACT specifically targets Ca^2+^-amplified glycolytic flux, thereby reducing lactate production without impairing basal glycolysis, enhancing ATP generation efficiency and ultimately restoring LSECs functionality (**Figure 4F**). Consistent with MICU1's context-dependent roles, liver-targeted siMicu1-LNPs potentiated (**Figure 7**) whereas LSECs-specific Micu1 overexpression driven by a Stab2 promoter AAV largely abolished (**Figure 8**) the hepatoprotective and sinusoid-stabilizing effects of ACT. Together, although MICU1-Ca^2+^ signaling is also relevant in hepatocytes and KCs, the role of ACT in acute HIRI primarily resides in LSECs. This phenomenon may involve the intensity and duration of Ca^2+^ signaling, warranting further in-depth investigation.

Epigenetic modifications, particularly histone lactylation, play a crucial role in maintaining cellular homeostasis and disease progression by regulating gene transcription. Under hypoxic stress conditions, lactate accumulation driven by glycolysis can induce lactylation that reshapes the epigenome 27, in which the metabolite lactate serves as a substrate for lactylation and is significantly involved in chronic liver diseases such as liver fibrosis and hepatocellular carcinoma 28, 29. However, the regulatory mechanism of lactylation in acute HIRI remains unclear. In this study, we discovered that HIRI-induced glycolysis significantly enhanced H3K18la modification through lactate accumulation, which was specifically enriched in the MICU1 promoter region. ACT inhibited lactate accumulation and ameliorated HIRI by directly suppressing H3K18la-mediated MICU1 transcriptional activation (**Figure 4** and **5**). Moreover, ACT exhibited selectivity in influencing the regulation of MICU1. At the initial stage of injury, ACT rapidly and directly binds to MICU1, thereby blocking Ca^2+^ transfer and restoring mitochondrial homeostasis. Additionally, ACT reshaped the metabolic microenvironment by inhibiting H3K18la modification to downregulate MICU1 transcription, fundamentally disrupting the vicious cycle of Ca^2+^ imbalance. The lactylation of non-histone proteins is also involved in gene regulation under HIRI 30. In the future, we will further investigate the specific mechanisms of non-histone protein lactylation in ACT's reversal of HIRI and restoration of LSECs function. It is noteworthy that liver macrophages and LSECs exchange metabolic intermediates through the perisinusoidal space. Under pathological conditions of aggravated inflammation in metabolic dysfunction-associated steatotic liver disease 31 and HCC 32, liver macrophages also exhibited the increased H3K18la levels similar to those in LSECs due to a lactate-accumulated microenvironment. Subsequently, we may focus on whether ACT also regulates macrophage function through H3K18la in future studies.

MCU-i4, as an inhibitor of MICU1, directly regulates the activity of the MCU complex and reduces Ca^2+^ influx into mitochondria. However, it may negatively impact mitochondrial membrane potential and inhibit muscle cell growth 33. Therefore, it is necessary to find safer and more effective inhibitors to regulate excessive Ca^2+^ influx. The C_26_/C_27_/C_40_/C_41_ hydroxyl groups of ACT were identified as key binding sites for MICU1 (S155, I156, T209, H315) (**Figure 8**). The removal of these groups (ACT-d2) resulted in the loss of its ability to regulate Ca^2+^ and glycolysis, while the derivative retaining the key hydroxyl group (ACT-d1) exhibited stronger MICU1 affinity and alleviated mitochondrial Ca^2+^ accumulation. This discovery provides a precise target for chemical modification in the development of MICU1-specific inhibitors to combat HIRI injury. Recently, the structure of the complete MCU-EMRE-MICU1-MICU2 complex has been resolved. The Lys/Arg loop of MICU1 could form a “cap” structure that stabilized its binding to the D261 loop of MCU through hydrogen bonds, hydrophobic/π-stacking interactions, and helix dipole interactions 34. Most recently, another natural ingratiate berberine was reported to directly bind to the juxtamembrane loop domain of MCU and disrupt MCU-EMRE assembly against mitochondrial Ca^2+^ overload 35. Although the key interaction site of MICU1 with MCU (Y114) differs from the sites where ACT interacts with MICU1 (S155, I156, T209, H315), further studies are necessary to determine whether hypoxic stress stimulates the conformational rearrangement of MICU1, preventing its binding to MCU, and whether ACT regulates Ca^2+^ by stabilizing the inhibitory dimer of MICU1 or further disrupting MICU1-MCU-EMRE assembly.

## 5. Conclusion

In summary, ACT competitively binds to MICU1, restoring mitochondrial Ca^2+^ homeostasis and reversing pathological glycolytic reprogramming through dual modulation of ER-mitochondrial Ca^2+^ flux and lactylation-dependent epigenetic circuitry. Our findings establish ACT as a potential MICU inhibitor, offering a safe therapeutic strategy for HIRI or diseases associated with dysregulated Ca^2+^ homeostasis between organelles.

## Supplementary Material

Supplementary materials and methods, figures and tables.

## Data Availability

Data will be made available on request.

## Abbreviations

ACT: acteoside
CUT&Tag: cleavage under targets & tagmentation
ECAR: extracellular acidification rate
ER: endoplasmic reticulum
FITC: fluorescein Isothiocyanate
FSA: formaldehyde-treated serum albumin
HIRI: hepatic Ischemia-reperfusion injury
HK: hexokinase
HR: hypoxia/re-oxygenation
IP3R: inositol 1,4,5-triphate receptor
LDH: lactate dehydrogenase
LNPs: lipid nanoparticles
LSECs: liver sinusoidal endothelial cells
MCU: mitochondrial calcium uniporter
MICU1: mitochondrial calcium uptake 1
MitoSOX: mitochondrial superoxide
OCR: oxygen consumption rate
PFK: phosphofructokinase
ROS: reactive oxygen species
SERCA2A: sarcoplasmic/ endoplasmic reticulum Ca (^2+^)ATPase 2a
Stab-2: stabilin 2
VDAC1: voltage dependent anion channel 1
