# Supplementary material for: Novel strategies for mitigating hepatic ischemia-reperfusion injury: targeting MICU1 and calcium-glycolytic homeostasis with acteoside
Source: Int J Biol Sci. 2026 Mar 30;22(8):4074–94. doi: 10.7150/ijbs.126332 (PMC13137855; doi:10.7150/ijbs.126332)
Supplement: Supplementary file 1 — Supplementary materials and methods, figures and tables. [file ijbsv22p4074s1.pdf]

Novel strategies for mitigating hepatic ischemia-reperfusion injury: targeting MICU1 and calcium-glycolytic homeostasis with acteoside

Yinhao Zhang [a], Runping Liu [b], Yun Yang [a], Huayao Lu [a], Mengyu Guo [a], Hong Wang [a], Ranyi Luo [a], Xiaoyong Xue [a], Guifang Fan [b], Kaihong Xie [a], Jia Liu [a], Xiaojiaoyang Li [a,*]

Table of contents

1. Supplementary materials and methods.. 2

2. Supplementary figures and legends.. 10

3. Supplementary table 25

1. Supplementary materials and methods

1.1 Materials

ACT (S31661), oxamate (OM, S30701) and 2-aminoethyl diphenylborinate (2-APB, S47898) were obtained from Yuanye Bio-Tech (Shanghai, China). Sodium L-lactate (S6010) was sourced from Selleck Chemicals LLC (Texas, USA). Galloflavin (GF, HY-W040118) and Rhod-2 AM (HY-D0989) were acquired from MCE (New Jersey, USA). Reactive oxygen species (ROS, O041), luciferase (RG005), L-lactate (S0204S), MitoSOX Red (S0061S), pyruvate, glucose (Glu, S0201S), Mito-Tracker red (C1049), Mito-Tracker green (C1048) and the D-lactate (S0204S) kits were purchased from Beyotime Biotechnology (Shanghai, China). These kits for detecting aspartate aminotransferase (AST, C010-2-1), alanine transaminase (ALT, C009-2-1), lactate dehydrogenase (LDH, A020-2-2), pyruvate kinase (PK, A076-1-1), hexokinase (HK, A077-3), phosphofructokinase (PFK, A129-1-1) were obtained from Nanjing Jiancheng Bioengineering Institute. The Seahorse XF Cell Mitochondria Stress Detection Kit (103010-100) was purchased from Seahorse (Boston, USA).

1.2 Bioinformatic analysis of HIRI-related genes

Bioinformatic analysis of HIRI-related genes from Gene Expression Omnibus (GEO) database. We selected the dataset GSE14951 (5 LT recipients), GSE112713 (11 LT recipients), GSE12720 (19 LT recipients), and performed the differential analysis of gene expression data in dataset by the GEO2R online tool in the GEO database. Only those genes with adjusted *P* value < 0.05 and $|\log_2\text{fold change}| \geq 0.5$

were considered differentially expressed genes (DEGs).

1.3 Transmission electron microscopy (TEM)

Liver tissue was fixed using an electron microscopy fixative and preserved at 4 °C. Subsequently, the tissue was washed with phosphate-buffered saline (PBS, 0.1 M, pH 7.4) and fixed in 1% osmium tetroxide (OsO_4) in 0.1 M PBS for 2 h at room temperature. The tissue was then dehydrated in ethanol and embedded in resin. The resin-embedded tissue was polymerized in an oven at 65 °C for 48 h and subsequently cut into 60 nm thin sections. These sections were examined using transmission electron microscopy (HT7800, Hitachi) to analyze the characteristics of the 'fenestrae' in LSECs.

1.4 Histological examination

The liver samples were immersed in 4% paraformaldehyde at 4°C for 24 h. Following dehydration through an alcohol gradient, clearing, and embedding in paraffin wax, the sliced sections (4 μm) were stained with hematoxylin and eosin (H&E) stains to assess the cell structure changes and inflammatory cell infiltration in different liver regions.

1.5 Immunofluorescence staining

LSECs were washed with PBS buffer, fixed in 4 % formaldehyde for 1 h, blocked and permeabilized with 1 % PBS-BSA with 0.1 % Triton-X100, followed by incubation with primary antibodies against Nogo and CKAP4 in a wet chamber at 4°C overnight. Later, cells were incubated with secondary antibodies and then counterstained with DAPI. All immunofluorescence images were captured by Olympus FV3000 confocal

laser scanning microscopy (Olympus, FV3000). For live cell staining experiments, LSECs were stained and photographed using Mito-Tracker Red CMXRos, DCFH-DA, ER-Tracker Green, MitoSOX Red and Rhod-2, following the dilution ratios and incubation times specified in the instructions. Fluorescence microscopy images were obtained using the Olympus FV3000 confocal laser scanning microscope.

1.6 Measurement of the extracellular acidification rates (ECAR) and oxygen consumption rate (OCR)

For measurement of ECARs and OCR in LSECs, we used the Agilent Seahorse XF cell glycolytic stress test kit and cell substrate oxidation stress test kit respectively, according to the manufacturer instructions. Initially, LSECs were inoculated (7,000/well) on the XF96 cell culture plate overnight and replaced culture medium with XF detection medium. Subsequently, we injected substrate mixture (glucose, oligomycin and 2-DG) to assess the glycolytic capacity of the LSECs. For the measurement of OCR, after replacing the cells with the detection solution similar to ECAR measurements, we evaluated basal respiration, maximal respiration, spare respiratory capacity and ATP-related respiration in LSECs through sequential injections of oligomycin, FCCP, a rotenone mixture, and antimycin A, respectively. All ECAR/OCR measurements were normalized to a seeding density of 7,000 cells per well.

1.7 ATP content measurement

The intracellular ATP content was measured using an ATP content kit (Beyotime Biotechnology, S0026). Following relative treatments, LSEC lysates were centrifuged

at 12,000 g for 5 min and quantified protein concentration using the BCA Protein Assay Kit. The LSEC samples were then mixed with the ATP detection solution for measuring the ATP content using a fluorescent microplate reader (Epoch 2TC, BIO-TEK, USA).

1.8 Co-immunoprecipitation

Briefly, after different treatments, 400 μL of pre-chilled lysis buffer was added to and lysed LSECs at 4°C for 30 min. Following this, the mixture was centrifuged at 10,000 g for 10 min at 4°C to transfer the supernatant to a clean 1.5 mL centrifuge tube. Subsequently, after quantifying the protein concentration by BCA assay, an aliquot of 800 μg of total cellular proteins were incubated with 6 μL of MCU monoclonal antibody at 4°C for 3 h. After this incubation period, 20 μL of Protein A/G PLUS Agarose (Thomo, 88804) were added and continued incubation overnight at 4°C on a rotating device. On the second day, we collected the immunoprecipitates, centrifuged them at 2,500 rpm for 5 min at 4°C, washed the pellet four times with 1.0 mL of PBS, and identified the proteins using Western blotting analysis.

1.9 Molecular docking

In this study, we employed a semi-flexible docking method to establish stable complexes. MICU1 (UniProt ID: Q8VCX5) was selected as the receptor, while ACT (PubChem ID: 5281800), modified ACT and calcium ions were chosen as the relative ligands. Molecular docking was conducted using AutoDock Vina version 1.1.2 software. Protein preprocessing, including the removal of water molecules and excess ligands, as well as the addition of hydrogen atoms, were performed using PyMOL 2.4. Energy

minimization of the compounds was carried out with ChemDraw 20.0. AutoDock Tools version 1.5.6 was utilized to generate PDBQT files for the docking simulations. The docking conformation exhibiting the lowest binding energy and highest clustering frequency was identified as the most promising binding mode between the ligand and the protein. Finally, the docking results were visualized using PLIP and PyMOL 2.4 software.

1.10 RNA isolation and quantitative real-time-PCR

The total RNA from LSECs were isolated by Trizol reagent and were reverse transcribed to cDNA. The expressions of the target genes were detected by SYBR Green quantitative real-time polymerase chain reaction. The mRNA expression of *Eif2ak3*, *Ddit3*, *Atf6*, *Atf4*, *Hspa5*, *Ern1*, *Vdac1*, *Atp2a2, Itpr1*, *Irf3*, *Gck*, *Pfkl*, *Pklr, Mcu, Micu1* and *Ldha* were calculated by their ratios to the housekeeping gene *Hprt1*. Further inquiries of primers for qPCR can be directed to the corresponding author.

1.11 Western blotting analysis

Proteins in LSECs and mouse livers were lysed and prepared with RIPA buffer obtained from BIORIGIN. Proteins were separated with SDS-PAGE gel and transferred to a polyvinylidene fluoride (PVDF) membrane (Merck Millipore, Darmstadt). Bands were incubated with the different primary antibodies at 4°C overnight and incubated with relative secondary antibodies for 1 h. The reaction products were visualized by ChemiDocTM Touch Imaging System (Bio-Rad, Hercules).

Additional information for involved antibodies and other details were provided in the Table S1.

1.12 LSECs uptake FITC-FSA assay

We prepared the FITC-FSA solution according to the previously established protocol [1]. Following HR stimulation and ACT treatment, we incubated FITC-FSA (100 μg/ml) with LSECs for 1 h. After staining the nuclei with DAPI, the uptake function of LSECs was assessed using confocal laser scanning microscopy (Olympus, FV3000).

1.13 Multiplex immunofluorescence staining

To examine changes in calcium channels on mitochondria-associated membranes (MAM), multiplex immunofluorescence staining was performed. We utilized a four-color multiplex immunofluorescence kit (Recordbio, RC0086-34) alongside antibodies targeting VDAC1 (66345-1-Ig, Proteintech), IP3R (19962-1-AP, Proteintech) and MCU (26312-1-AP, Proteintech). The staining process involved incubating these antibodies with horseradish peroxidase-conjugated secondary antibodies, followed by tyramide signal amplification (TSA) and finally staining the nuclear DNA using DAPI. All multiplex immunofluorescence images were captured by Leica TCS SP8 X confocal laser scanning microscope (Leica, Germany).

1.14 Cellular thermal shift assay (CETSA)

LSECs were incubated with DMSO or 50 μM ACT for 0.5 h and then washed 3 times with PBS. Cells were then suspended in 1 ml PBS, divided into equal volumes,

and heated at different temperatures (ranging from 37°C to 70°C) for 3 min. After heating, three rapid freeze-thaw cycles were applied to lyse cells, followed by Western blotting analysis.

1.15 Drug affinity responsive target stability (DARTS)

We used M-PER supplemented with protease and phosphatase inhibitors to lyse LSECs and then determined the protein concentration of the cell lysates using the BCA protein assay. Lysates containing 500 μg of total protein were incubated with DMSO or 50 μM ACT for 1 h at room temperature, and digested for 30 min using pronase at dilutions of 1:300, 1:1,000, 1:2,000, 1:3,000 and 1:10,000. Immediately after digestion, the reaction was terminated by adding protein loading buffer and heating. The samples were then analyzed by Western blot.

1.16 Chromatin immunoprecipitation-quantitative polymerase chain reaction (ChIP-qPCR)

9 × 10^6 LSECs were seeded on 15 cm plates and treated with either HR or ACT. The LSECs were cross-linked with 1% formaldehyde for 10 min, followed by a cross-linking was quenched by adding glycine to a final concentration of 0.125 M for 5 min at room temperature. Subsequently, LSECs were washed three times with ice-cold PBS and then lysed in lysis buffer (50 mM Tris-HCl, pH 8.0, 10 mM EDTA, 1% SDS) for 10 minutes on ice. Then, we utilized a JY92-IIN ultrasonic cell disrupter to sonicate the cells (9 W, 10 s each time, four times in total). Finally, we carried out immunoprecipitation using the anti-H3K18la antibody (PTM-1427RM, Jingjie PTM

BioLab) and quantified the precipitated DNA fragments containing the *Mcu, Micu1* and *Vdac1* promoters using qPCR.

1.17 Luciferase reporter assay

Stab2 is represented as a scavenger receptor and is preferentially expressed in LSECs. To investigate whether the Stab2 promoter can be utilized to construct AAV viruses targeting LSECs, we performed a luciferase reporter assay on the Stab2 promoter. We cloned the mouse wild-type (WT) Stab2 promoter (2144 bp) and two truncated variants [Stab2-1 promoter (644 bp, spanning from -500 to +144), Stab2-2 promoter (1144 bp, spanning from -1000 to +144)] into the pGL3 basic vector. The firefly luciferase gene was inserted downstream of the cloned promoter sequences in the pGL3 basic vector and co-transfected with an internal control plasmid into 293T cells for 36 h. The luciferase reporter gene activity was then measured according to the manufacturer's instructions for the Firefly Luciferase Reporter Gene Assay Kit (Beyotime, China).

2. Supplementary figures and legends

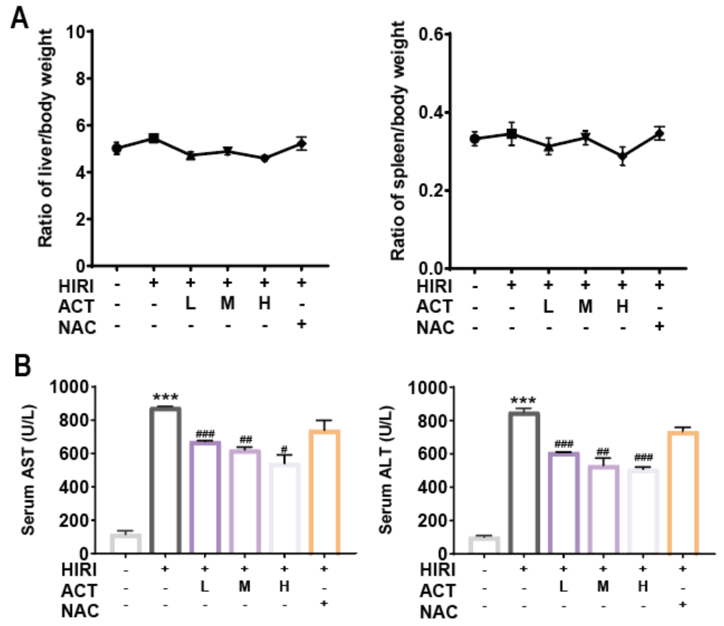

Figure S1. ACT alleviates the hepatic function injured by HIRI process (**A**) Body weight and organ coefficients. (**B**) Serum AST, ALT levels. Data are presented as mean ± SEM. ****P* < 0.001, compared to the Sham group; #*P* < 0.05, ##*P* < 0.01, ###*P* < 0.001, compared to the HIRI group by two-way ANOVA (**B**). ACT, acteoside; NAC, N-acetylcysteine.

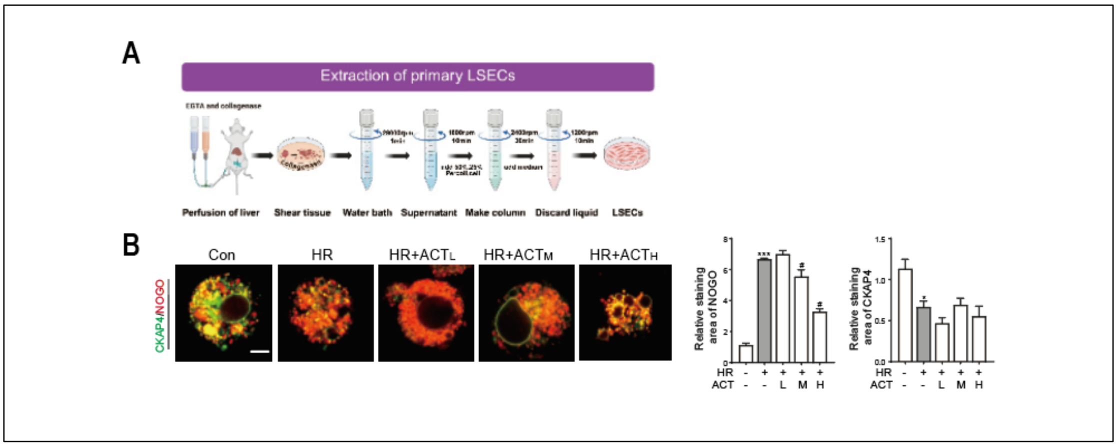

Figure S2. ACT restores ER-mitochondrial homeostasis in HR-stimulated LSECs

(**A**) The workflow of extraction of primary LSECs from mouse livers. (**B**) Representative images of CKAP4 and NOGO in LSECs (left panel), and quantitative analysis of CKAP4 and NOGO staining intensity (right panel), scale bar = 20 μm. Data are presented as mean ± SEM. **P* < 0.05, ****P* < 0.001, compared to the con group; #*P* < 0.05, compared to the HR group by two-way ANOVA. ACT, acteoside; HR, hypoxia and reoxygenation.

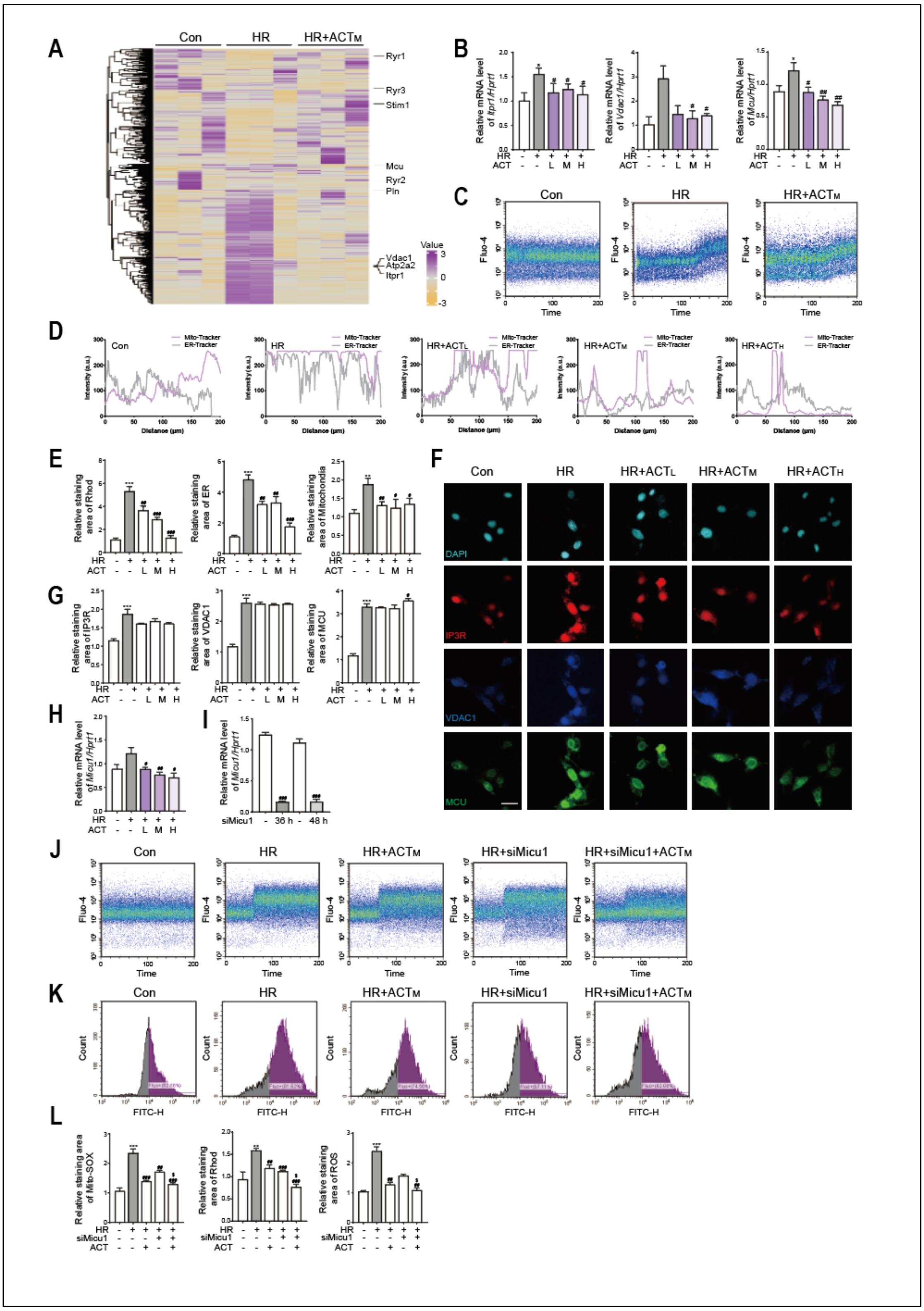

Figure S3. ACT inhibits the Ca^{2+} transfer to mitochondrial *via* MICU1 (**A**) The heatmap of Ca^{2+} signaling pathways in LSECs. (**B**) The mRNA levels of *Itpr1*, *Vdac1*,

Mcu and normalized with *Hprt1* in LSECs. (**C**) The Ca^{2+} transient frequency in LSECs. (**D**) ER-mitochondria colocalization analysis in LSECs. (**E**) The statistical charts of Rhod-2, ER-tracker and mito-tracker in LSECs. (**F**) The images of IP3R, VDAC1 and MCU single channel staining in LSECs, scale bar = 20 μm. (**G**) The statistical charts of IP3R, VDAC1 and MCU in LSECs. (**H**) The mRNA levels of *Micu1* and normalized with *Hprt1* in LSECs. (**I**) The knockdown efficiency of siMicu1 in LSECs. (**J**) The Ca^{2+} transient frequency treated with si-*Micu1* in LSECs. (**K**) The intracellular Ca^{2+} levels in LSECs treated with si-*Micu1* in LSECs. (**L**) The statistical charts of mito-SOX, Rhod and ROS treated with si-*Micu1* in LSECs. Data are presented as mean ± SEM. $^{*}P < 0.05$, $^{**}P < 0.01$, $^{***}P < 0.001$, compared to the con group; $^{\#}P < 0.05$, $^{\#\#}P < 0.01$, $^{\#\#\#}P < 0.001$, compared to the HR group; $^{\$}P < 0.05$, compared to the relative HR + siMicu1 group by two-way ANOVA by two-way ANOVA (**B**, **E**, **G**, **H**, **I**, **L**). ACT, acteoside; ER, endoplasmic reticulum; HR, hypoxia and reoxygenation; Mito, mitochondria; ROS, reactive oxygen species.

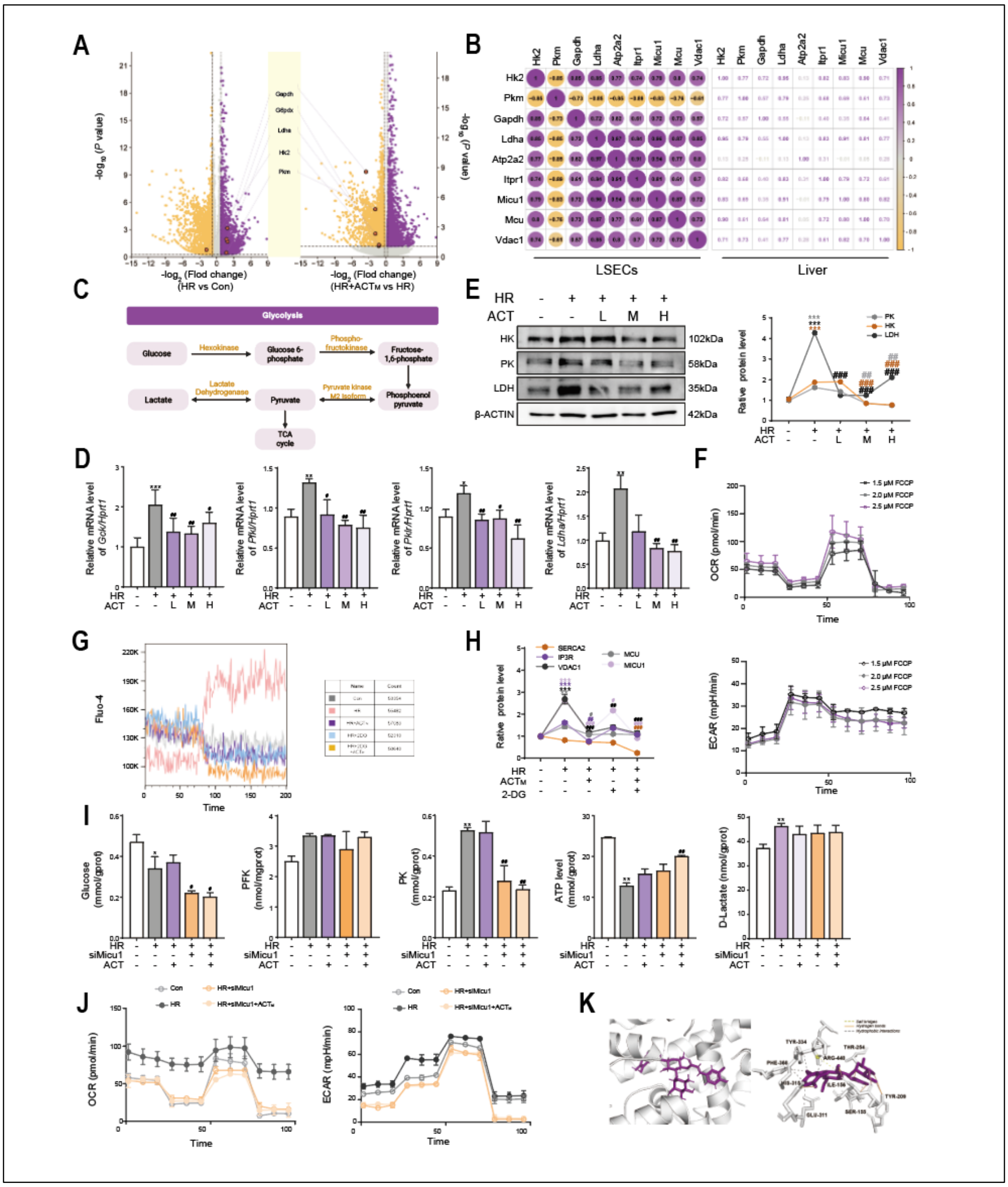

Figure S4. ACT directly binding to MICU1 to inhibit Ca^{2+}-stimulated glycolysis (**A**) Biphasic volcano plot of glycolysis-related genes in LSECs. (**B**) Correlation heatmap of glycolytic processes gene expression profiles in LSECs and liver. (**C**) Schematic diagram of the glycolysis process. (**D**) The mRNA levels of *Gck*, *Pfkl*, *Pklr*, *Ldha* and normalized with *Hprt1* in LSECs. (**E**) The protein levels of HK, PK, LDH and β-ACTIN

in LSECs. (**F**) Confirmation of FCCP concentrations in OCR and ECAR. (**G**) Ca^{2+} transient amplitude of LSECs treated with 2-DG. (**H**) The quantitative statistics of SERCA2, IP3R, VDAC1, MCU and MICU1 protein level in LSECs normalized by β-ACTIN. (**I**) The levels of glucose, PFK, PK, ATP, D-lactate in LSECs treated with si-*Micu1*. (**J**) The OCR and ECAR of LSECs treated with si-*Micu1*. (**K**) Molecular docking of MICU1 and ACT. Data are presented as mean ± SEM. $^{*}P < 0.05$, $^{**}P < 0.01$, $^{***}P < 0.001$, compared to the con group; $^{\#}P < 0.05$, $^{\#\#}P < 0.01$, $^{\#\#\#}P < 0.001$, compared to the HR group by two-way ANOVA (**D**, **E**, **H**, **I**). 2-DG, 2-Deoxy-D-glucose; ACT, acteoside; ATP, adenosine triphosphate; FITC, fluorescein Isothiocyanate; FSA, formaldehyde-treated serum albumin; HK, hexokinase; HR, hypoxia and reoxygenation; LDH, lactate dehydrogenase; PFK, phosphofructokinase; PK, pyruvate kinase; TCA, tricarboxylic acid cycle.

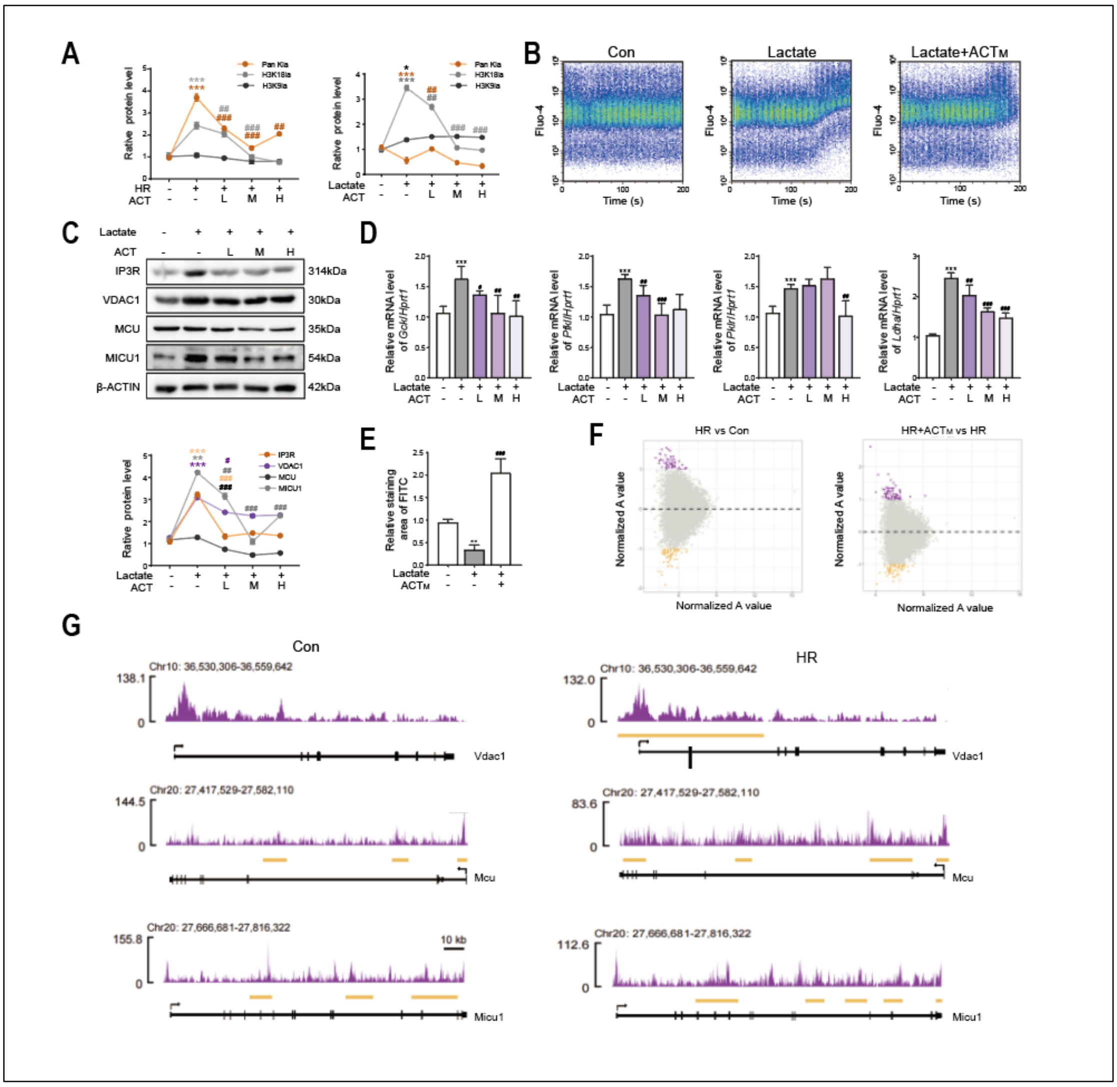

Figure S5. ACT ameliorates HR-induced metabolic dysfunction in LSECs through H3K18la (**A**) The quantitative statistics of global lactylation, H3K18la and H3K9la protein level in LSECs normalized by β-ACTIN. (**B**) The Ca^{2+} transient frequency of LSECs. (**C**) The protein levels of IP3R, VDAC1, MCU, MICU1 and β-ACTIN in LSECs. (**D**) The mRNA levels of *Gck*, *Pfkl*, *Pklr*, *Ldha* and normalized with *Hprt1* in LSECs. (**E**) The statistical charts of FITC-FSA in LSECs. (**F**) MA plot of differentially expressed genes. (**G**) H3K18la peaks in the promoter regions of *Vdac1*, *Mcu* and *Micu1*. Data are presented as mean ± SEM. *P < 0.05, **P < 0.01, ***P <

0.001, compared to the con group; $^{\#}P < 0.05$, $^{\#\#}P < 0.01$, $^{\#\#\#}P < 0.001$, compared to the HR group (**A**, **C**, **D**, **E**). ACT, acteoside; FITC, fluorescein Isothiocyanate; HR, hypoxia and reoxygenation.

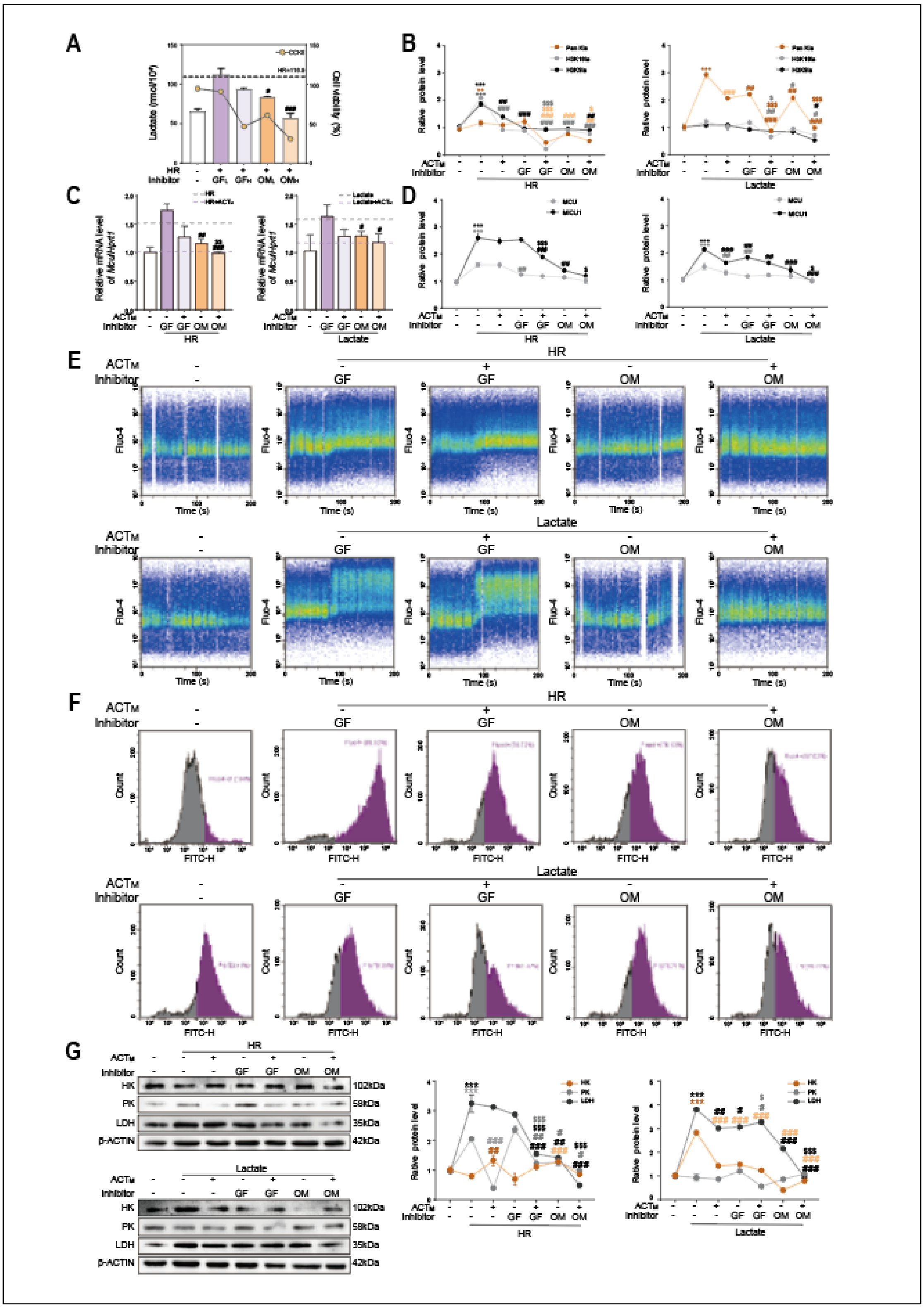

Figure S6. ACT and lactate inhibitors synergistically suppress Ca^{2+} transfer and glycolytic processes in LSECs (**A**) Dynamic detection of cell viability and lactate

content of LSECs treated with galloflavin and oxamate. (**B**) The quantitative statistics of global histone lactylation, H3K18la and H3K9la protein levels in LSECs normalized by β-ACTIN. (**C**) The mRNA levels of *Mcu* normalized with *Hprt1* in LSECs. (**D**) The quantitative statistics of MCU and MICU1 protein levels in LSECs normalized by β-ACTIN. (**E**) The Ca^{2+} oscillation kinetics (**F**) and intracellular Ca^{2+} levels in LSECs treated with galloflavin and oxamate. (**G**) The protein levels of HK, PK and LDH protein levels in LSECs normalized by β-ACTIN. Data are presented as mean ± SEM. $^{**}P < 0.01$, $^{***}P < 0.001$, compared to the con group; $^{\#}P < 0.05$, $^{\#\#}P < 0.01$, $^{\#\#\#}P < 0.001$, compared to the relative model (HR or lactate) groups; $^{\$}P < 0.05$, $^{\$\$}P < 0.01$, $^{\$\$\$}P < 0.001$, compared to the relative model (HR or lactate) + inhibitor (GF or OM) groups by two-way ANOVA (**A**, **B**, **C**, **D**, **G**). ACT, acteoside; GF, galloflavin; HK, hexokinase; HR, hypoxia and reoxygenation; LDH, lactate dehydrogenase; OM, oxamate; PK, pyruvate kinase.

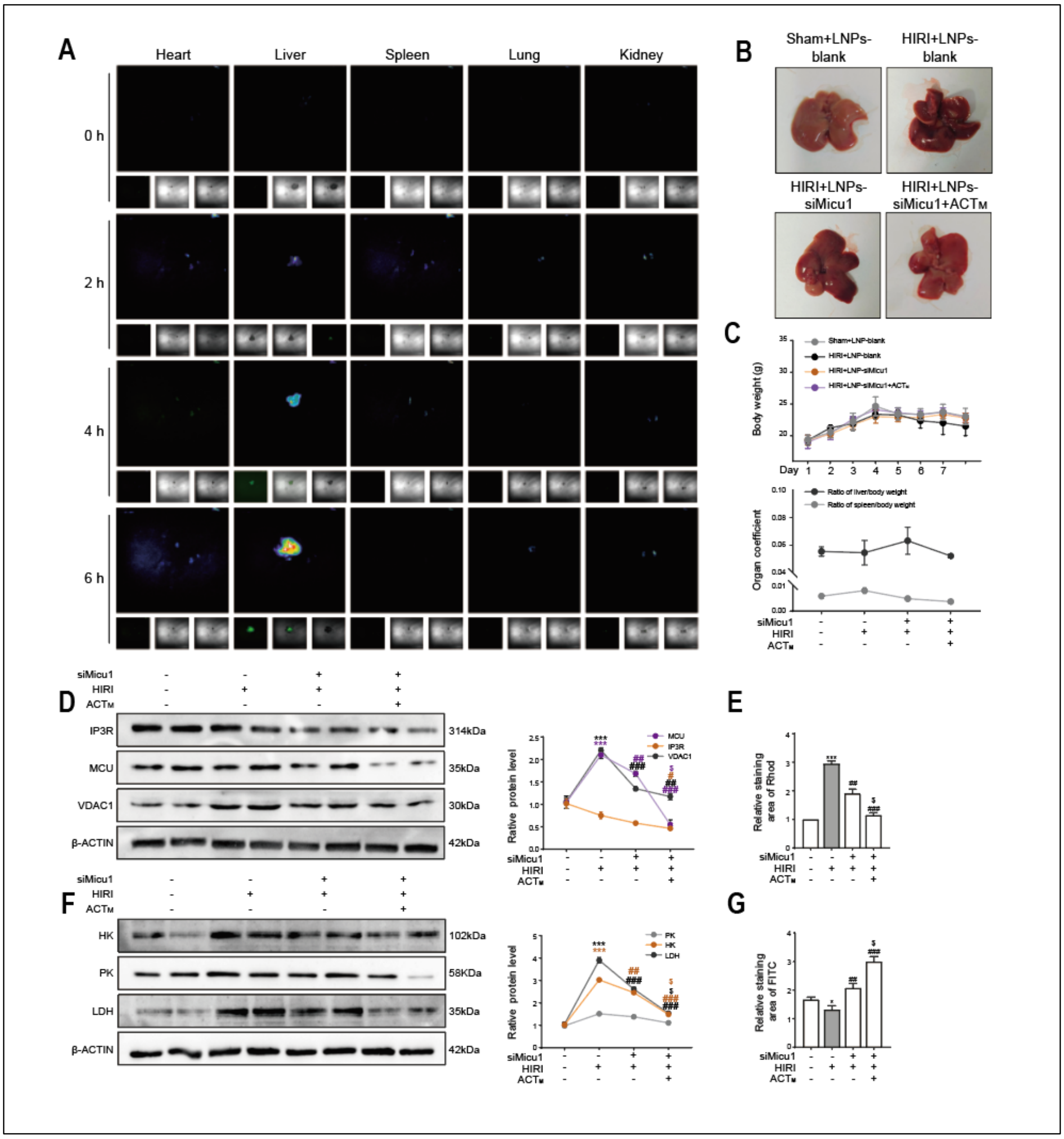

Figure S7. The target localization of LNPs, changes in liver morphology and weight, Ca^{2+}-glycolysis-related protein changes, Rhod and FITC fluorescence intensity in ACT and LNPs-siMicu1 treated mice (**A**) Time-course biodistribution of siMicu1-LNPs by *in vivo* imaging system at 0, 2, 4 and 6 h post-injection. (**B**) Liver images in different groups. (**C**) Body weight and organ coefficients. (**D**) The protein levels of IP3R, VDAC1 and MCU protein levels in LSECs normalized by β-ACTIN. (**E**) The statistical charts of Rhod-2 in liver. (**F**) The protein levels of HK, LDH and PK

protein levels in LSECs normalized by β-ACTIN. (**G**) The statistical charts of FITC-FSA in liver. Data are presented as mean ± SEM. *P < 0.05, **P < 0.01, ***P < 0.001, compared to the Sham group; $^{\#}P$ < 0.05, $^{\#\#}P$ < 0.01, $^{\#\#\#}P$ < 0.001, compared to the HIRI group; $^{\$}P$ < 0.05, compared to the HIRI + LNPs-siMicu1 groups by two-way ANOVA (**D**, **E**, **F, G**). ACT, acteoside; FITC, fluorescein Isothiocyanate; HK, hexokinase; LDH, lactate dehydrogenase; LNPs, lipid nanoparticles; PK, pyruvate kinase.

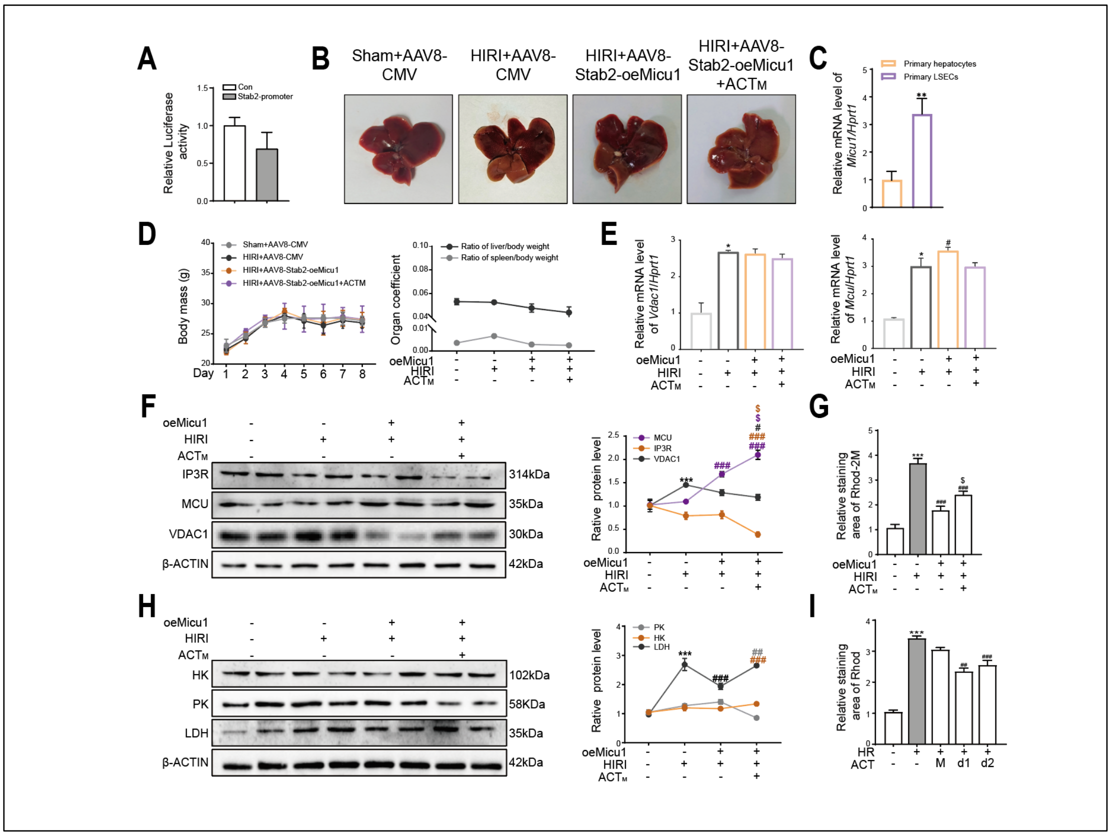

Figure S8. LSECs-specific *Micu1* overexpression exacerbates HIRI even with the presence of ACT by promoting Ca^{2+} delivery and glycolytic processes (**A**) Dual-luciferase activity of truncated Stab2 promoter in AML12 cells. (**B**) Liver images in different groups. (**C**) Detection of *Micu1* mRNA expression in primary hepatocytes and LSECs isolated from oeMicu1 mice. (**D**) Body weight and organ coefficients. (**E**) The mRNA levels of *Vdac1* and *Mcu* normalized with *Hprt1* in liver. (**F**) The protein levels of IP3R, VDAC1 and MCU protein levels in LSECs normalized by β-ACTIN. (**G**) The statistical charts of Rhod-2M in liver. (**H**) The protein levels of HK, PK and LDH protein levels in LSECs normalized by β-ACTIN. (**I**) The statistical charts of Rhod-2M in LSECs. Data are presented as mean ± SEM. **P* < 0.05, ***P* < 0.01, ****P* < 0.001, compared to the Sham group; #*P* < 0.05, ##*P* < 0.01, compared to the HIRI group; $*P* < 0.05,

compared to the HIRI + AAV8-Stab2-oeMicu1 groups by two-way ANOVA (**E**, **F**, **G**, **H**, **I**). AAV8, adeno-associated virus serotype 8; ACT, acteoside; HK, hexokinase; LDH, lactate dehydrogenase; PK, pyruvate kinase.

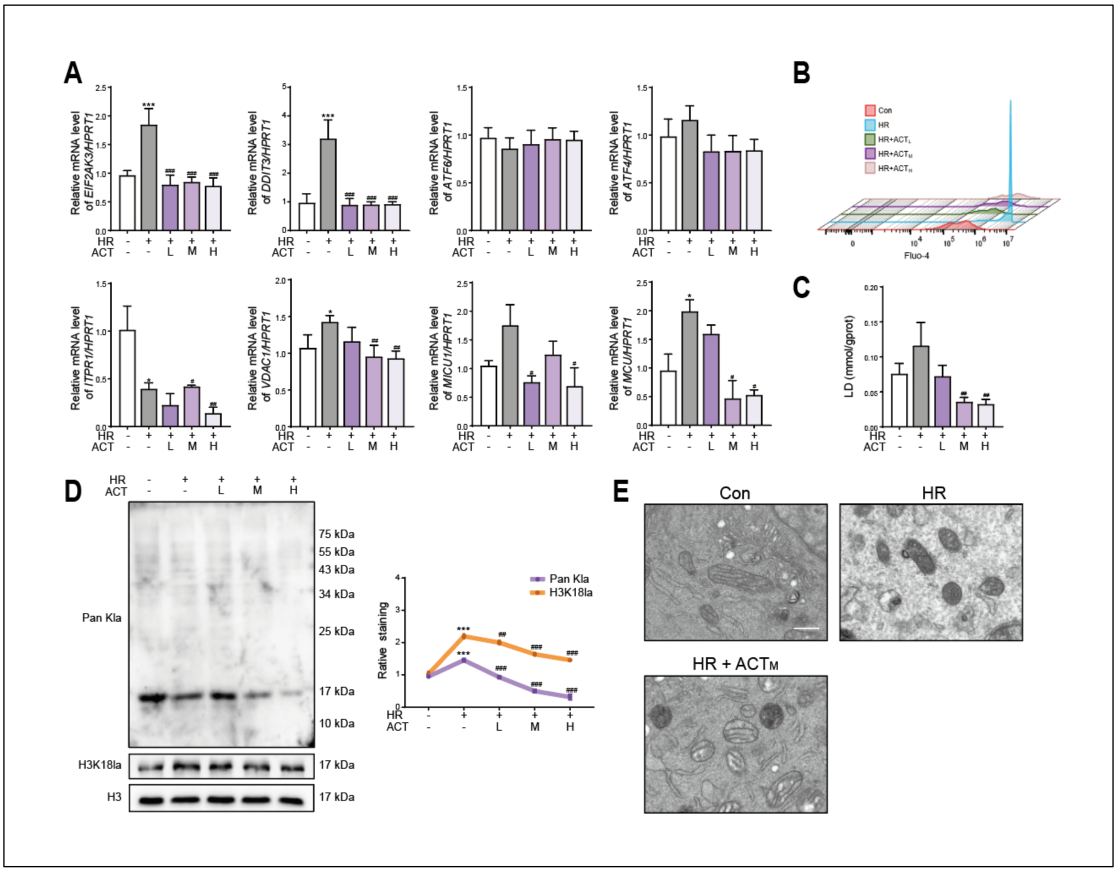

Figure S9. ACT ameliorates HR-induced ER stress-Ca^{2+} dysregulation-H3K18la regulatory cascade in human LSECs (**A**) Relative mRNA expression levels of *EIFAK3*, *DDIT3*, *ATF4*, *ATF6*, *ITPR1*, *VDAC1*, *MCU*, *MICU1* and normalized with *HPRT1* in human LSECs. (**B**) Intracellular Ca^{2+} levels in human LSECs. (**C**) Lactate content of human LSECs. (**D**) Representative images and quantitative statistics of global lactylation and H3K18la protein level in human LSECs normalized by β-ACTIN. (**E**) TEM analysis of human LSECs. Data were presented as mean ± SEM (n= 3 for each group). $^{*}P < 0.05$, $^{***}P < 0.001$, compared to the con group; $^{\#}P < 0.05$, $^{\#\#}P < 0.01$, $^{\#\#\#}P < 0.001$, compared to the HR group by two-way ANOVA (**A**, **C** and **D**). ACT, acteoside; HR, hypoxia and reoxygenation; LD, lactate; TEM, transmission electron microscopy.

3. Supplementary table

Table S1. The information of used antibodies.

Name	Abbreviation	Dilution	Supplier	Cat no.
SERCA2, ATP2A2 Polyclonal antibody	SERCA2	1:4000	Proteintech	27311-1-AP
GRP78/BIP Monoclonal antibody	GRP78	1:4000	Proteintech	66574-1-Ig
IRF3 Monoclonal antibody	IRF3	1:1000	Proteintech	66670-1-Ig
ITPR1-specific Polyclonal antibody	IP3R	1:1000	Proteintech	19962-1-AP
MCU/CCDC109A Polyclonal antibody	MCU	1:2000	Proteintech	26312-1-AP
CBARA1 Polyclonal antibody	MICU1	1:2000	Proteintech	25905-1-AP
Beta Actin Monoclonal antibody	β-ACTIN	1:5000	Proteintech	66009-1-Ig
Histone H3 Polyclonal antibody	H3	1:1000	Proteintech	17168-1-AP
CKAP4 Polyclonal antibody	CKAP4	1:1000	Proteintech	16686-1-AP

Hexokinase 2 Polyclonal antibody	HK	1:4000	Proteintech	2029-1-AP
PKM2-specific Polyclonal antibody	PK	1:4000	Proteintech	15822-1-AP
LDHB Polyclonal antibody	LDH	1:4000	Proteintech	14824-1-AP
Anti-L-Lactyl Lysine Rabbit mAb	Pan Kla	1:2000	PTM BIO	PTM-1401RM
Anti-L-Lactyl-Histone H3 (Lys9) Rabbit mAb	H3K9la	1:1000	PTM BIO	PTM-1419RM
NOGO (C-4)	NOGO	1:500	SantaCruz	sc-271878
PERK (B-5)	PERK	1:500	SantaCruz	sc-377400
VDAC1/Porin (B-6)	VDAC1	1:500	SantaCruz	sc-390996

Reference:

1. Jia K, Zhang Y, Luo R, Liu R, Li Y, Wu J, et al. Acteoside ameliorates hepatic ischemia-reperfusion injury via reversing the senescent fate of liver sinusoidal endothelial cells and restoring compromised sinusoidal networks. Int J Biol Sci. 2023; 19: 4967-88.
